# Multi-site tumor sampling highlights molecular intra-tumor heterogeneity in malignant pleural mesothelioma

**DOI:** 10.1186/s13073-021-00931-w

**Published:** 2021-07-14

**Authors:** Clément Meiller, François Montagne, Theo Z. Hirsch, Stefano Caruso, Julien de Wolf, Quentin Bayard, Jean-Baptiste Assié, Léa Meunier, Yuna Blum, Lisa Quetel, Laure Gibault, Ecaterina Pintilie, Cécile Badoual, Sarah Humez, Françoise Galateau-Sallé, Marie-Christine Copin, Eric Letouzé, Arnaud Scherpereel, Jessica Zucman-Rossi, Françoise Le Pimpec-Barthes, Marie-Claude Jaurand, Didier Jean

**Affiliations:** 1 Centre de Recherche des Cordeliers, Inserm UMRS-1138, Sorbonne Université, Université de Paris, Functional Genomics of Solid Tumors, Paris, France; 2 Present address: Service de Chirurgie Thoracique, Hôpital Calmette, CHRU de Lille, Lille, France; 3 Present address: Service de Chirurgie Thoracique et Transplantation Pulmonaire, Hôpital Foch, Suresnes, France; 4 University Paris-Est Créteil (UPEC), CEpiA (Clinical Epidemiology and Ageing), EA 7376- IMRB, UPEC, Créteil, France; 5 GRC OncoThoParisEst, Service de Pneumologie, CHI Créteil, UPEC, Créteil, France; 6 Programme Cartes d’Identité des Tumeurs (CIT), Ligue Nationale Contre Le Cancer, Paris, France; 7 Present address: IGDR UMR 6290, CNRS, Université de Rennes 1, Rennes, France; 8 Assistance Publique-Hôpitaux de Paris, Hôpital Européen Georges Pompidou, Paris, France; 9 Service d’Anatomopathologie et Cytologie, Université de Paris, Hôpital Européen Georges Pompidou, Paris, France; 10 Univ. Lille, CHU Lille, Service de Chirurgie Thoracique, Hôpital Calmette, Lille, France; 11 Univ. Lille, CHU Lille, Institut de Pathologie, Lille, France; 12 Univ. Lille, CNRS, Inserm, CHU Lille, Institut Pasteur de Lille, UMR9020 – UMR1277 - Canther – Cancer Heterogeneity, Plasticity and Resistance to Therapies, Lille, France; 13 Centre National Référent MESOPATH, Centre Leon Berard, Lyon, France; 14 Present address: Département de Pathologie Cellulaire et Tissulaire, CHU d’Angers, Angers, France; 15 Univ. Lille, CHU Lille, Service de Pneumologie et d’Oncologie Thoracique, unité INSERM 1189 OncoThAI, Lille, France; 16 Réseau National Expert pour le Mésothéliome Pleural Malin (NETMESO), Lille, France; 17 Service de Chirurgie Thoracique, Hôpital Européen Georges Pompidou, Paris, France

**Keywords:** Thoracic tumor, Spatial molecular intra-tumor heterogeneity, Clonality, *NF2* subclonal mutation, Tumor microenvironment

## Abstract

**Background:**

Malignant pleural mesothelioma (MPM) is a heterogeneous cancer. Better knowledge of molecular and cellular intra-tumor heterogeneity throughout the thoracic cavity is required to develop efficient therapies. This study focuses on molecular intra-tumor heterogeneity using the largest series to date in MPM and is the first to report on the multi-omics profiling of a substantial series of multi-site tumor samples.

**Methods:**

Intra-tumor heterogeneity was investigated in 16 patients from whom biopsies were taken at distinct anatomical sites. The paired biopsies collected from apex, side wall, costo-diaphragmatic, or highest metabolic sites as well as 5 derived cell lines were screened using targeted sequencing. Whole exome sequencing, RNA sequencing, and DNA methylation were performed on a subset of the cohort for deep characterization. Molecular classification, recently defined histo-molecular gradients, and cell populations of the tumor microenvironment were assessed.

**Results:**

Sequencing analysis identified heterogeneous variants notably in *NF2*, a key tumor suppressor gene of mesothelial carcinogenesis. Subclonal tumor populations were shared among paired biopsies, suggesting a polyclonal dissemination of the tumor. Transcriptome analysis highlighted dysregulation of cell adhesion and extracellular matrix pathways, linked to changes in histo-molecular gradient proportions between anatomic sites. Methylome analysis revealed the contribution of epigenetic mechanisms in two patients. Finally, significant changes in the expression of immune mediators and genes related to immunological synapse, as well as differential infiltration of immune populations in the tumor environment, were observed and led to a switch from a hot to a cold immune profile in three patients.

**Conclusions:**

This comprehensive analysis reveals patient-dependent spatial intra-tumor heterogeneity at the genetic, transcriptomic, and epigenetic levels and in the immune landscape of the tumor microenvironment. Results support the need for multi-sampling for the implementation of molecular-based precision medicine.

**Supplementary Information:**

The online version contains supplementary material available at 10.1186/s13073-021-00931-w.

## Background

Malignant pleural mesothelioma (MPM) is a rare and highly aggressive tumor arising in the thoracic cavity. Exposure to asbestos is the main risk factor, and despite the ban of this mineral fiber in several countries, MPM remains a major public health problem worldwide. In most patients, MPM is an incurable cancer with a very poor prognosis, notably due to the ineffectiveness of conventional anti-tumor treatments. The reference treatment is based on systemic platinum-based chemotherapy combined with pemetrexed, a treatment that improves survival by only a few months even with the recent addition of the anti-VEGF therapy bevacizumab [1]. More recently, immunotherapy based on immune checkpoint inhibitors has shown survival benefits in particular in patients with non-epithelioid histology, without an accurate predictive biomarker for treatment response besides age and histology [2–4]. Despite this recent therapeutic progress, there is still an urgent need to develop a precision medicine approach taking into account MPM heterogeneity.

Like most solid tumors, MPM is a heterogeneous cancer with high variability between patients [5]. The heterogeneity of MPM was first described at the histologic level by defining three main types: epithelioid, sarcomatoid, and biphasic. Different histologic subtypes have been described especially for the epithelioid type including, but, not limited to, tubulopapillary, acinar, trabecular, solid, and micropapillary architectural subtypes [6, 7]. Interestingly, studies show that histologic subtype and grade of epithelioid MPM have a prognostic impact [6, 8]. More recently, the rare transitional type, which could also be a subtype of the sarcomatoid type, was described [9]. Large-scale omics and next-generation sequencing (NGS) studies have demonstrated MPM inter-tumor heterogeneity at the molecular level and led to molecular classifications into two to four subtypes [10–13]. More recent publications have shown that MPM heterogeneity is well-described by a continuum [10, 14]. Recently, we defined histo-molecular gradients, which also takes into consideration intra-tumor heterogeneity, by determining the proportions of epithelioid-like and sarcomatoid-like cellular entities (E.score and S.score), related to the two extreme histological types of MPM, within tumor samples. These gradients have a high prognostic value and are of interest to guide therapeutic strategies, including targeted therapies and immunotherapies [10].

MPM is a solid tumor characterized by a diffuse locoregional growth within the pleural cavity. A polyclonal origin has been described and subclonal cell populations with specific mutations have been evidenced [15, 16]. So far, only two studies have focused on spatial genetic heterogeneity [17, 18]. Variability in the mutational load without involving key genes of mesothelial carcinogenesis was first observed between anatomical locations in a series of six patients [17]. Very recently, a series of nine patients also highlighted heterogeneous mutations between anatomical locations [18]. Some evidence supports spatial heterogeneity concerning the tumor microenvironment [5]. Both the above studies showed distinct T-cell repertoires depending on the tumor anatomic region [17, 18]. However, contrary to inter-tumor microenvironmental heterogeneity in terms of stromal and immune cell infiltration [10, 19], intra-tumor cell heterogeneity of the tumor microenvironment has not yet been reported.

In the present study, we have gone further in the characterization of MPM molecular spatial heterogeneity in the largest series of patients (16 cases) studied so far. We not only defined the heterogeneity at the genetic level and, for the first time, characterized the spatial dysregulation of gene expression, epigenetic changes, and tumor microenvironment differential infiltration.

## Methods

### Patients

Tumor samples were collected from up to four distinct anatomical sites (apex, side wall, costo-diaphragmatic, and highest metabolic sites detected by positron emission tomography (PET) scan when present, shown in Additional file 1: Figure S1) in a series of 16 patients who had diagnostic biopsies or surgery resections for MPM in two French hospitals (CHRU of Lille and Hôpital Européen Georges Pompidou of Paris). For each patient, the tumor location is indicated (A: apex; B: side wall; C: costo-diaphragmatic; D: highest metabolic site). Non-tumoral samples (blood or muscle of the chest wall) were available for 9 patients. Patients were diagnosed between 2014 and 2017 and tumors were certified by the French National Pathology Expertise Network (Mesopath) as MPM [20]. Histologic type, subtype, and grade were determined by MesoPath expert pathologists and part of the series was reviewed according to the WHO 2021 update [21]. The experiments were undertaken with the understanding and written consent of each subject. The study methodologies were conformed to the standards set by the Declaration of Helsinki and approved by a local medical ethics committee (CPP Ile-de-France II). The sampling procedures were approved by the French research ministry (CODECOH no. DC-2016-2771). Samples were annotated with detailed clinico-pathological and epidemiologic information obtained from pathology reports (Additional file 1: Table S1). Based on tumor purity and a quality check of extracted nucleic acids, two tumor samples per patient were used for further analysis. Three metrics were used to evaluate tumor purity: (i) pathologist assessment of tumor cellularity, (ii) prediction by RT-qPCR of non-tumor components using WISP deconvolution method [10], and (iii) frequencies of variants determined by targeted NGS. For the few patients with more than 2 qualified tumor samples, paired samples with the closest tumor purity were kept. In addition, MPM primary cell lines were generated from five patients in our laboratory, from fresh tumor samples collected at sites distinct from the four previous locations. They were established, cultured as previously described [22], and used at low-passage numbers (6 to 10 passages). All tumor samples and cell lines were analyzed by targeted NGS, 9 paired tumor samples by whole exome sequencing (T199LE-A/B, T200LE-A/C, T201LE-A/B, T203LE-A/C, T225LE-A/D, T227LE-A/D, T277HP-A/C, T278HP-A/C, T333HP-A/C), 8 by RNA sequencing (T199LE-A/B, T201LE-A/B, T203LE-A/C, T225LE-A/D, T227LE-A/D, T277HP-A/C, T278HP-A/C, T333HP-A/C), and 5 by methylation profiling (T203LE-A/C, T227LE-A/D, T277HP-A/C, T278HP-A/C, T333HP-A/C).

### Nucleic acid extraction

For tumor samples, a preliminary step of tissue disruption and cell lysis was achieved using TissueLyser II (Qiagen, Courtaboeuf, France). Genomic DNA and total RNA from tumor samples and cell pellets, obtained from cultures of MPM primary cell lines at passages 6 to 10, were extracted using the AllPrep DNA/RNA/miRNA Universal kit (Qiagen) according to the manufacturer’s protocol. For the fresh tumor sample used to establish MPM_83, automated DNA extraction was performed on multiple sections (*n* = 34) of the frozen preserved sample following the protocol of the Maxwell 16 Tissue DNA purification kit and the Maxwell instrument (Promega, Charbonnières-les-Bains, France). DNA and RNA quantifications were done by fluorescence measurements (Hoechst dye) on a FLUOstar Omega microplate reader (BMG Labtech, Champigny sur Marne, France) and by spectrometry on a NanoDrop-1000 (Ozyme, Saint-Cyr-l’Ecole, France), respectively. We used agarose gel migration for DNA to ensure the absence of excessive degradation before sequencing and methylation analysis. We assessed RNA integrity on a Fragment Analyzer (Agilent Technologies, Courtaboeuf, France) before RNA-seq analysis.

### Targeted next-generation sequencing (NGS) and variant calling

We performed targeted NGS using our in-house protocol recently published in detail to sequence 21 genes and the *TERT* promoter on the whole series of samples (16 patients) [22, 23]. Briefly, library preparation was based on a multiplex PCR enrichment and sequencing was achieved by a MiSeq instrument (Illumina, Evry, France). FASTQ files were generated by the Illumina MiSeq Reporter software. Primer sequences were removed using the fastx_trimmer function from the fastx toolkit. Reads were aligned on the human genome assembly hg19/GRCh37 using the Burrows-Wheeler aligner (BWA) and bam files were generated using samtools. Variant calling was performed with Unified Genotyper and variant annotation was obtained using the Oncotator annotation algorithm, along with the ensembl Variant Effect Predictor (VEP) algorithm and the Annovar annotation. Finally, based on these annotations, all somatic variants with a functional consequence were checked by visualization using Integrative Genomic Viewer (IGV) software (Broad Institute, Cambridge, MA, USA). Variants of interest were validated either using whole exome sequencing (WES) data or by a second independent targeted NGS. Genome coordinates were converted to human genome assembly hg38/GRCh38 using the UCSC LiftOver online tool [24].

### Whole exome sequencing (WES) and variant calling

Library preparation, exome capture, sequencing, and data analysis were performed by IntegraGen (Evry, France). We screened all paired biopsies with non-tumoral samples available (9 patients) [23]. Briefly, genomic DNA was captured either using the Agilent in-solution enrichment methodology with their biotinylated oligonucleotide probe library (SureSelect Clinical Research Exome V2, Agilent Technologies) or the Twist Human Core Exome Enrichment System (Twist Bioscience, San Francisco, USA), followed by paired-end 75 base massively parallel sequencing on Illumina HiSeq4000. Sequence capture, enrichment, and elution were performed according to the manufacturer’s instructions and protocols without modification, except for the library prepared using NEBNext® Ultra II kit (New England Biolabs, Evry, France). Image analysis and base calling were performed using Illumina Real-Time Analysis (2.7.7) with default parameters. Sequence reads were mapped to the human genome build hg38/GRCh38 by using the BWA tool. The duplicated reads were removed using sambamba tools. Variant calling, allowing the identification of genetic alterations, as well as SNV (single nucleotide variation) and small insertions/deletions (up to 20 bp), was performed via the Broad Institute’s GATK Haplotype Caller GVCF tool (3.7) for constitutional DNA and via the Broad Institute’s MuTect tool for somatic DNA. Ensembl’s VEP (variant effect predictor) program processed variants for further annotation [25, 26]. An in-house post-processing workflow was applied to filter out candidate somatic mutations that were more consistent with artifacts or germline mutations. Finally, the variants were validated by visualization using IGV software. Two bioinformatics predictions for missense pathogenicity were used: SIFT (5.2.2) and PolyPhen (2.2.2) and damaging variants were considered if it was predicted by at least one tool. The circular binary segmentation algorithm implemented in the Bioconductor package DNAcopy (DNAcopy 1.32.0) as well as FACET R package (v.0.6.1.) were used to reconstruct copy-number profiles from WES data [27, 28]. For FACET analysis, single nucleotide polymorphisms (SNP) count matrix for both tumoral and non-tumoral samples was obtained by processing bam files with snp-pileup (arguments: -q15; -Q20; -P100; -r20,0). Then, SNP matrix was processed using preProcSample function (cval = 25, snp.nbhd=250, ndepth=30) to generate log-R-ratio and the segmentation. ProcSample (cval=150, min.nhet=5) was then used to estimate the wild-type 2-copy state. Finally, emcncf (min.nhet=5) function was used to estimate sample ploidy and purity. R package ggplot was used to make pangenomic graphical representations. Cancer-related genes were based on Tier 1 of the Cancer Gene Census (CGC) database [29, 30].

### Clonality prediction

For each mutation, we used the Palimpsest R package [31] to estimate the fraction of tumor cells harboring this variant (cancer cell fraction, CCF) in each tumor, as previously described [32]. In addition, the emcncf (min.nhet=5) function of FACET package (v.0.6.1.) was used to determine cellular fraction and allele-specific copy-numbers per segments from CNV data [28]. T200LE was excluded from this analysis as results were not reliable probably due to low tumor purity. CCF for CNV was calculated by dividing a cellular fraction by tumor purity. For pairs of tumor samples from a given patient, the comparison of the CCF distribution between samples was used to sort mutations or CNV among clonal shared, subclonal shared, clonal private, and subclonal private variants. Variants or CNV were considered subclonal if the CCF value of both tumor samples were lower than 0.5 and, for variants only, if the upper limit of the confidence interval was below 1. Shared variants were found in both paired tumor samples in contrast to private variants.

### RNA sequencing

Library preparation and sequencing were performed by IntegraGen on paired biopsies of 8 patients [23]. Libraries were prepared using the TruSeq Stranded mRNA kit (Illumina) or the NEBNext Ultra II Directional RNA Library Prep Kit (New England Biolabs), according to the supplier’s recommendations. Briefly, the key steps of both protocols were (i) purification of PolyA-containing mRNA molecules using poly-T oligo attached magnetic beads from 1 μg total RNA, (ii) RNA fragmentation using divalent cations at an elevated temperature to obtain approximately 300 bp fragments, (iii) double-strand cDNA synthesis, and (iv) Illumina adapter ligation and cDNA library amplification by PCR for sequencing. Paired-end 75 base massively parallel sequencing was then carried out on an Illumina HiSeq4000. FASTQ files were aligned to the human reference genome hg38/GRCh38 using TopHat2 V.2.0.14 [33]. We removed reads mapping to multiple locations, and we used HTSeq [34] to obtain the number of reads associated with each gene in the Gencode v27 database. The Bioconductor DESeq2 package [35] was used to import raw HTSeq counts for each sample into R statistical software (R Foundation for Statistical Computing, Vienna, Austria) and to apply variance stabilizing transformation to the raw count matrix. FPKM values (number of fragments per kilobase of exon model and millions of mapped reads) were calculated by normalizing the count matrix for the library size estimated with the DESeq2 package and the coding length of each gene. Differentially expressed genes were determined using both FPKM and DESeq2-normalized RNA-seq data: genes that displayed a FPKM value below 1 in both paired biopsies were excluded and we considered as differentially expressed only genes with a difference of DESeq2 expression values between paired samples above 1. Hierarchical clustering was done using cosine distance and Ward’s linkage method in R statistical software with the 500 most differentially expressed genes. Network analysis of the distribution of the differentially expressed genes among tumor samples was performed using the web-based interactive tool DiVenn [36].

### Gene fusion detection

Fusions detected by TopHat2 (--fusion-search --fusion-min-dist2000 --fusion-anchor-length13 --fusion-ignore-chromosomes chrM) were filtered using the TopHatFusion pipeline [37]. We discarded artifacts based on fusion redundancy among samples from different patients. Fusions validated by BLAST and with at least 20 split-reads or pairs of reads spanning the fusion event in at least one of the paired tumor samples were retained. Shashimi plots were generated using junctions.bed provided by TopHat2 to determine the junctions supported by splitted reads and bedtools genomecov function (with -bga -split options) to calculate the coverage from the aligned bam files.

### Molecular classification and histo-molecular score predictions

Expression data from RNA sequencing (DESeq2-normalized counts) were used to predict molecular subgroups and histo-molecular scores (E/S.score) based on centroids and deconvolution approaches, respectively, as described previously [10].

### Methylation profiling

Experimental works and data analysis were performed by IntegraGen on paired biopsies of 5 patients [38]. First, bisulfite conversion of 500 ng of DNA samples was performed with the Zymo EZ DNA Methylation Kit (Ozyme) according to the manufacturer’s instructions. Then, methylation analysis was performed on the Infinium MethylationEPIC BeadChip Kit (Illumina) following the supplier’s recommendations. Briefly, samples were denatured, neutralized, and incubated in the Illumina hybridization oven for 20–24 h at 37 °C to uniformly amplify genomic DNA, then enzymatically fragmented. Following the fragmentation step, samples were hybridized to the BeadChip and incubated at 48 °C for 16–24 h in the Illumina hybridization oven. Finally, non-hybridized and unspecific hybridized DNA samples were washed from the BeadChip, and labeled nucleotides were added to extend and stain primers hybridized to the samples. The Illumina iScan system (with the iScan Control Software) was used to scan Illumina MethEpic BeadChips and raw data were analyzed with GenomeStudio software v2011 according to Illumina’s recommendations to generate result files with beta-values. Loci were considered as differentially methylated between paired tumor samples when both detection *p*-values were lower than 0.05 and a difference of at least 0.2 was observed between beta-values of each tumor sample. To compare with genes previously reported to be correlated to the E.score or the S.score [10], we only considered genes with expression changes in the expected sense according to the histo-molecular scores of the paired biopsies. Hierarchical clustering was done using cosine distance and Ward’s linkage method in R statistical software with the 500 CpG showing the most variability in their methylation beta-value.

### Pathway dysregulation analysis

Signal pathway dysregulation analysis was performed by two complementary approaches : over-representation analysis and single-sample Gene Set Enrichment Analysis (ssGSEA). Differentially expressed or methylated genes lists obtained for each patient were used as input for over-representation analysis in the web-based Gene SeT AnaLysis Toolkit using the KEGG, Reactome, or GeneOntology Biological Process non-redundant databases [39]. We only considered pathways with false discovery rate (FDR) adjusted *p*-values lower than 0.05. Gene ratio is the proportion of differentially expressed genes regarding the total number of genes included in a given pathway. SsGSEA were calculated as gene-set variation analysis (GSVA) enrichment scores using the DESeq2-normalized RNA-seq data, after excluding genes with a FPKM value below 1 for all samples, and the GSVA package [40]. The absolute value of the delta of each ssGSEA score between paired tumor samples (ssGSEA_score) was used to assess intra-tumor heterogeneity.

### Tumor microenvironment estimation

The microenvironment cell population counter (MCP-counter) method [41] was used to compute scores of infiltration for different cell populations from DESeq2-normalized RNA-seq data. To optimize the prediction of the relative abundance of stromal and immune cells within MPM tumors, the genes included in the signature of each cell population were selected based on their absence of expression in 22 MPM cell lines. *IGKC* and *CHRM3-AS2* were also excluded as gene expression was not present in all datasets. The complete list of genes used for each population is available in Additional file 1: Table S2. For integrative analysis with public datasets, a total of 295 samples from three different series of RNAseq were combined in the integrated analysis, including the 16 paired tumor samples, 209 samples from Bueno et al. [11, 42], and 70 samples from TCGA [13] downloaded through the Broad Institute TCGA GDAC firehose tool [43]. Then, we standardized gene expression separately to have a mean of 0 and a standard deviation of 1 per gene in each dataset. Statistical analysis and data visualization were performed using R software. Unsupervised hierarchical clustering was performed using cosine distance and Ward’s linkage method. The Wilcoxon test was used to estimate the difference in the abundance of immune populations within paired tumor samples.

### Immunohistochemical staining

Formalin-fixed, paraffin-embedded (FFPE) tumor biopsies (3 biopsies at distant sites per patient) were sectioned at a thickness of 3 μm and stained on positively charged glass slides. Deparaffinization, rehydration, and antigen retrieval were performed by CC1 (prediluted; pH 8.0) antigen retrieval solution (Ventana Medical Systems, Inc.) on the Ventana BenchMark ULTRA automated slide stainer for 32 min at 100 °C. Specimens were incubated with primary antibodies anti-CD3 (clone LN10; Leica; dilution 1:100), anti-CD8 (clone C8/144B; Dako, dilution 1:25), and anti-CD20 (clone L26; Diagomics; dilution 1:150) followed by visualization with the Ultraview DAB IHC Detection Kit. The specimens were then counterstained with hematoxylin II and bluing reagent (Ventana) and coverslipped. Each IHC run contained a positive control.

## Results

### Mutational intra-tumor heterogeneity

**Fig. 1:**
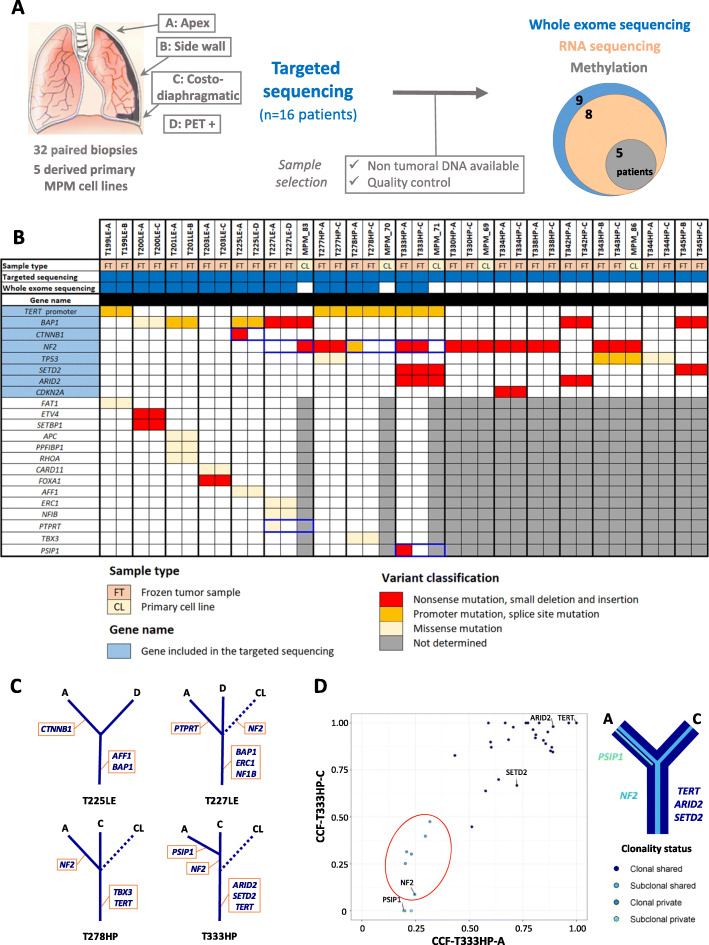
Study workflow and mutational intra-tumor heterogeneity. **a** Study workflow presenting the anatomical sites selected for the multi-sampling procedure and related molecular analysis showing the patient series size and sample selection. **b** Heatmap of the cancer-related gene variants with damaging consequences detected in paired biopsies and derived primary cell lines. The legend in the bottom left indicates the color codes used in the heatmap. Intra-tumor heterogeneous variants are framed in blue. **c** Mutational intra-tumor heterogeneity. Trees schematically illustrate the cancer-related gene variants with damaging consequences detected in paired biopsies and derived primary cell lines of the four patients displaying intra-tumor heterogeneity at the genetic level. Solid lines are for paired biopsies and dotted lines for primary cell lines (CL). **d** Clonality of the variants with structural consequences detected in tumor samples from patient T333HP. On the left, the graph shows the adjusted cancer cell fraction (CCF) values of each protein variant, validated using IGV visualization. Each variant was then categorized according to its clonality (clonal when present in all cells of a sample) as well as to its spatial segregation (shared when present in the two biopsies, private otherwise) and was colored according to its clonality status. Cancer-related genes are indicated. Subclonal variants only present in a fraction of cancer cells but in both paired biopsies are surrounded by a red circle and might be consistent with the polyclonal diffusion of these tumors throughout the thoracic cavity. On the right, the clonal evolution is schematically represented as a tree. For subclonal populations, the line width is proportional to the number of variants

Paired tumor samples collected prior to chemotherapy treatment at two distinct anatomical sites from 16 MPM patients and five MPM-derived primary cell lines were analyzed by different NGS and omics methods (Fig. 1a). Patient characteristics are detailed in Additional file 1: Table S1. In summary, patients were chemonaïve, mostly male (81%), had a past exposure to asbestos (62%), and were diagnosed at a median age of 74 years. One tumor was biphasic and the others were epithelioid, the most frequent histologic type. Even if the epithelioid tumors belong to different subtypes and grades, our series do not fully reflect the histologic diversity of MPM. Germline mutations in cancer-related genes for 9 patients are reported in Additional file 1: Table S3A. Among genes previously reported as altered by pathogenic or likely pathogenic germline mutations in MPM [44], damaging variants were found in *SDHA* and *PALB2* genes in patients T225LE and T277HP, respectively.

To investigate mutational intra-tumor heterogeneity, we performed targeted NGS on a panel of key genes of mesothelial carcinogenesis [22] for the entire series and WES for patients with non-tumoral DNA available to go deeper in their genetic characterization. Most of the protein-altering somatic variants, validated using multiple sequencing approaches and IGV visualization, were common between paired samples from a given patient (Fig. 1b, c; Additional file 1: Table S3B-D). However, we found intra-tumor heterogeneous variants in the well-known mesothelioma driver gene *NF2*. Among eight patients harboring *NF2* mutations, three displayed intra-tumor heterogeneity. The first patient (T227LE) had a *NF2* nonsense variant in the derived primary cell line (MPM_83), although it was not called in the paired biopsies. Therefore, we investigated the presence of the variant in the fresh tumor sample used to establish MPM_83 by sequencing multiple sections of this sample and found the *NF2* variant at different frequencies, confirming the subclonal status of the *NF2* mutation (Additional file 2: Figure S2, Additional file 1: Table S3E). For the second patient (T278HP), we detected an *NF2* splice site variant in the sample localized at the apex but it was absent in the paired costo-diaphragmatic sample and in the derived primary cell line (MPM_70). Finally, the third patient (T333HP) presented an *NF2* nonsense variant at low frequency in both biopsies, but the mutation was not found in the derived primary cell line (MPM_71), suggesting that this mutation was subclonal and probably absent in the fresh tumor sample used to establish MPM_71. In addition to *NF2*, we also detected intra-tumor heterogeneous mutations in three cancer-related genes [29]: (i) a frameshift insertion variant in *CTNNB1* for patient T225LE, (ii) a missense variant in the protein tyrosine phosphatase receptor *PTPRT* for patient T227LE (however, based on RNA-seq data, this gene does not seem to be expressed in both samples of this tumor), and (iii) a nonsense variant in the transcriptional coactivator *PSIP1* for patient T333HP, to our knowledge the first mutation in this gene described for MPM (Fig. 1b).

The clonality of the protein-altering variants was also assessed to investigate subclonal tumor populations that could be in different proportions among paired samples. To perform a robust analysis of clonality, we calculated the cancer cell fraction of each variant, by normalizing variant frequencies with copy number and tumor purity (Additional file 1: Table S4A). As expected, we found most of the cancer-related gene variants to be clonal and shared by both paired samples except for the four genes mentioned previously (Additional file 2: Figure S3). Tumor clonality was heterogeneous between patients, who showed a variable number of private variants ranging from 0 to 12 (Additional file 1: Table S4B). Strikingly, we found a subclonal tumor population present in both biopsies in patient T333HP supported by six variants, including *NF2* (Fig. 1d). Of note, the variant frequencies in the clonal tumor population (*ARID2*, *SETD2*, and *TERT* promoter) and in the subclonal population (*NF2*) were validated by targeted NGS for this patient (Additional file 1: Table S3B). This suggests the polyclonal dissemination of these tumors throughout the thoracic cavity. Furthermore, shared subclonal variants were also identified in three other patients, i.e., T199LE, T203LE, and T225LE (Additional file 2: Figure S3).

### Copy number variations and fusion transcripts

Because MPM is often characterized by chromosomal alterations, we took advantage of WES coverage and RNA-seq data to analyze copy number variations (CNV) and fusion transcripts, respectively. First, we found that pangenomic CNV between paired samples were very similar using both DNAcopy or FACET tools, notably in the *CDKN2A*, *BAP1*, and *NF2* loci (Additional file 2: Figures S4 and S5). Segmentation and CNV clonality provided by FACET analysis identified mainly clonal shared CNV, but also some clonal private (*n* = 22), subclonal private (*n* = 4), and subclonal shared (*n* = 1) CNV (Additional file 1: Table S4C). However, all homozygous deletions were clonal and shared by paired tumor samples, and, as expected, often located in the *CDKN2A* locus. Interestingly, we identified a CNV with a subclonal status in both paired tumor biopsies in patient T333HP, supporting previous results based on variant clonality analysis. Then, we searched for fusion transcripts in RNA-seq data that we generated for the same series of samples screened with WES, except for tumor samples from patient T200LE due to poor RNA quality in addition to very low tumor content. In three patients, we detected fusions involving a cancer-related gene, such as *STK11* and *KDM6A*, quoted as altered in MPM in the Cosmic database (release v91), or *TFG* reported in fusions in other cancers (Additional file 1: Table S5, Additional file 2: Figure S6A) [45]. Interestingly, we found that the fusion transcript in patient T277HP involving *TFG* and *ADGRG7* led to abnormal gene expression of the adhesion G-protein coupled receptor *ADGRG7*, a gene not commonly expressed in MPM (Additional file 2: Figure S6B). For these three fusions, both paired samples harbored the rearrangement. To note, we also detected four fusion transcripts restricted to one of the paired samples in three patients, but they did not involve cancer-related genes. In conclusion, we did not observe structural intra-tumor heterogeneity at the genomic level with an evident contribution to cancer development.

### Differential gene expression and signal pathway dysregulation

Gene expression analysis, from the RNA-seq data of paired samples from eight patients, indicated global gene expression as being patient-specific, as shown by unsupervised clustering (Additional file 2: Figure S7). The number of differentially expressed (DE) genes between paired samples ranged from 647 to 2119 genes, regardless of tumor cellularity (Spearman correlation test, R squared=0.01, *p* = 0.79), with a majority of protein coding genes (Additional file 2: Figure S8A). A high proportion of these protein coding genes was found to be differentially expressed in several tumor samples (Additional file 1: Table S6A, Additional file 2: Figure S8B). However, shared DE genes were distributed throughout all tumor samples (Additional file 2: Figure S8C). We performed signal pathway over-representation analysis using the KEGG, Reactome, and GeneOntology databases on protein coding genes differentially expressed between paired samples. Among the top recurrent pathways detected in all these databases, we found two main families, the first one related to cell adhesion and extracellular matrix organization and the second to immune communication (Additional file 1: Table S6B-D).

We also analyzed signal pathway dysregulation by ssGSEA across the whole cohort (Additional file 1: Table S6E). Recurrent major changes between paired tumor samples concern immune pathways, more precisely related to immunological synapses involving specific immune cells infiltration such as antigen-presenting cells (dendritic and B cells) and T cells.

### Histo-molecular heterogeneity

**Fig. 2:**
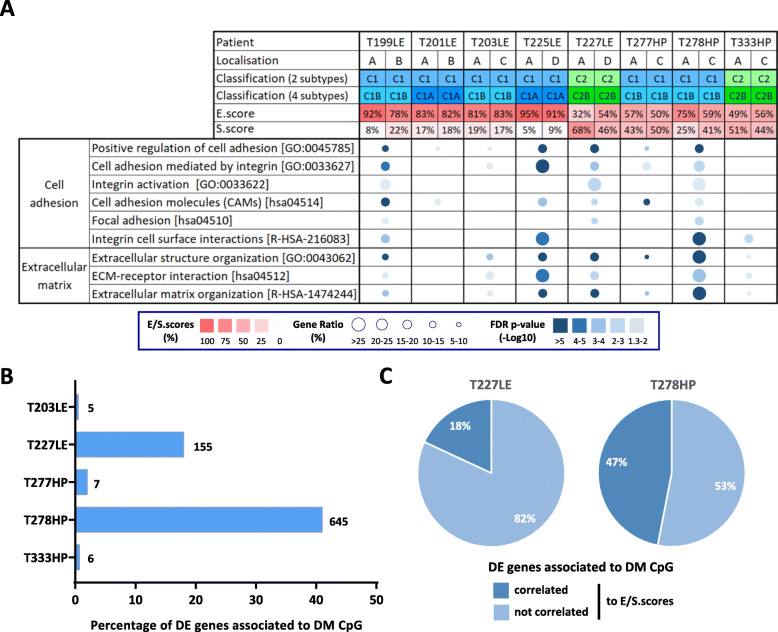
Intra-tumor heterogeneity at the transcriptomic and epigenetic levels. **a** Molecular heterogeneity (molecular classifications and histo-molecular gradients) of the paired tumor samples and the over-represented pathways linked to cell adhesion and the extracellular matrix are shown in the table. For each patient, the tumor location is indicated (A: apex; B: side wall; C: costo-diaphragmatic; D: highest metabolic site). The molecular classifications into two to four subtypes and the E/S.scores were predicted based on RNA-seq data and are colored in blue or green depending on the subtypes and with a red gradient for the E/S.scores. Pathway over-representation is indicated by a circle with the size and a color proportional to the gene ratio and the FDR *p*-values, respectively. **b**, **c** Based on transcriptome and methylome analysis, the differentially expressed protein coding genes with an associated differentially methylated CpG (DE_DM genes) between paired tumor samples were determined. The percentage of DE_DM genes among all the differentially expressed protein coding genes is shown in the histogram for each patient. The number of DE_DM genes is indicated at the right of the histogram bars (**b**). The proportion of protein coding genes previously shown to be correlated to the E/S.scores [10] in all DE_DM genes is indicated in the pie charts for patients T227LE and T278HP (**c**). DE: differentially expressed; DM: differentially methylated

We previously proposed classification in four molecular subtypes and histo-molecular gradients determining the proportion of epithelioid-like and sarcomatoid-like components (E/S.scores) to describe MPM inter- and intra-tumor heterogeneity [10]. Based on RNA-seq data, we first predicted the molecular subtypes and obtained similar subgroups between paired biopsies (Fig. 2a). Then, we estimated the E/S.scores and observed changes greater than 10% in the E/S.scores among paired samples in the three patients T199LE, T227LE, and T278HP. Interestingly, these three patients showed differential enrichment for the pathways associated with cell adhesion and extracellular matrix (Fig. 2a), which makes sense in the context of our previous publication [10]. As expected, patients T201LE, T203LE, T277HP, and T333HP displayed similar E/S.scores between paired samples and did not show major changes in these pathways. In contrast, patient T225LE showed a slight increase in the S.score, but a differential expression of genes enriched in these pathways. ssGSEA data also show a globally higher variation of these pathways between paired tumor samples of patients T225LE, T227LE, and T278HP and to a lesser extent T199LE compared to the four others (Additional file 2: Figure S9).

### Epigenetic intra-tumor heterogeneity

To evaluate intra-tumor heterogeneity at the epigenetic level, we performed methylome analysis in five patients with sufficient remaining quantities of DNA. As for the transcriptome analysis, the global methylation profile, illustrated by unsupervised clustering, remained very specific for each patient (Additional file 2: Figure S10A). Differential methylation analysis between paired samples showed that the majority of differentially methylated (DM) CpG were associated with protein coding gene loci in all patients (Additional file 1: Table S7A, Additional file 2: Figure S10B). However, this analysis highlighted that the number of DM CpG between paired samples varied by patient, with two patients (T227LE and T278HP) showing a high number of DM CpG, 5476 and 19178 respectively (Additional file 2: Figure S10C). Taking into account protein coding gene expression, a potential impact of methylation was only observed in these two patients, as DM CpG were identified in 18% and 41%, respectively, of DE genes (Fig. 2b). Interestingly, among the DE protein coding genes associated to DM CpG, 18% and 47%, respectively, in patients T227LE and T278HP, are genes previously identified to be correlated with the E/S.scores of the histo-molecular gradients (Fig. 2c). Furthermore, in T278HP, these genes were enriched in previously identified pathways involved in cell adhesion and extracellular matrix organization (Additional file 1: Table S7B; Additional file 2: Figure S10D). These results support the notion that epigenetic regulation could contribute to the variations occurring in the proportions of epithelioid-like and sarcomatoid-like components in patient T278HP and to a lesser extent in patient T227LE, in agreement with our previous studies demonstrating that histo-molecular gradients are related to epigenetic regulation [10].

### Tumor microenvironment

**Fig. 3:**
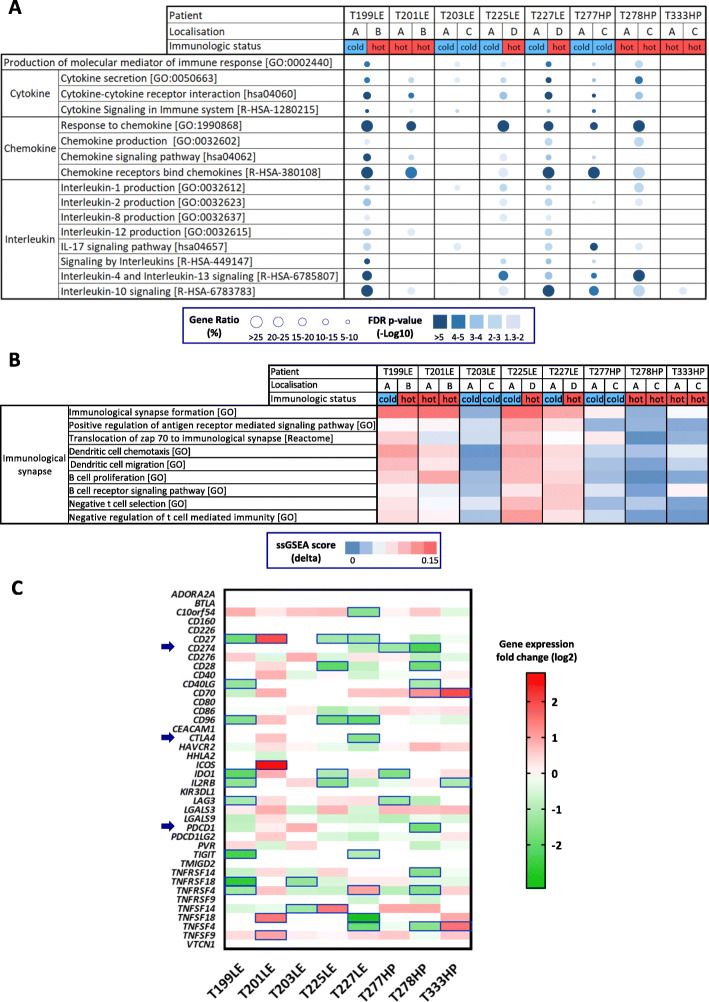
Intra-tumor heterogeneity of immune pathway. **a**, **b** Dysregulated immune pathways identified by over-representation analysis (**a**) and single-sample Gene Set Enrichment Analysis (ssGSEA) (**b**). For each patient, the tumor location is indicated (A: apex; B: side wall; C: costo-diaphragmatic; D: highest metabolic site). The immunologic status “hot” or “cold” was determined based on clusterization of stromal and immune cell infiltration (see Fig. 4). Over-representation of each pathway linked to immune communication is indicated as a circle, whose size is proportional to the gene ratio and the color gradient represents the FDR *p*-values (**a**). The differences in the ssGSEA score (delta_score) of each paired tumor sample are indicated as a color gradient (**b**). **c** The differential expression between paired tumor samples of immune checkpoints is shown in the heatmap. Differential expression was set to 0 for genes which did not display an FPKM score higher than 1 in at least one of the paired biopsies. The differentially expressed genes with a fold change of at least 2 are framed in blue. The genes encoding PDL1 (*CD274*), CTLA4 (*CTLA4*), and PD1 (*PDCD1*) are indicated by a blue arrow

**Fig. 4:**
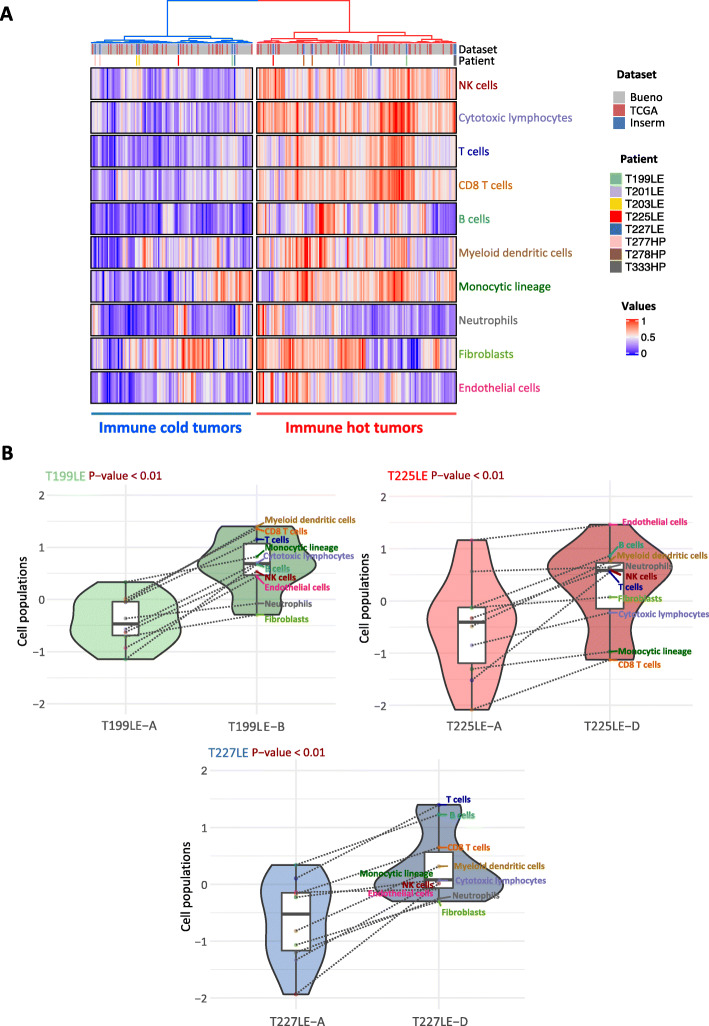
Differential infiltration of stromal and immune cell populations. **a** Unsupervised clustering of the paired tumor samples of eight patients with 209 and 70 tumor samples from the Bueno and TCGA series, respectively, was performed based on cell populations determined by the MCP-counter method. The paired tumor samples of the eight patients (series U1138) are indicated by a color code at the top of the heatmap as well as the series of each tumor sample. **b** The violin plots show the normalized MCP-counter values of immune and stromal cell populations between paired tumor samples for patients T199LE, T225LE, and T227LE, which are characterized by a hot/cold mixed immune profile. Each cell population is indicated in the box plots by a color point connected by a dotted line between paired tumor samples. The *p*-values of the Wilcoxon test comparing distribution between paired tumor samples are indicated at the top of the violin plot

We next analyzed the immune landscape changes in paired samples. Comparison based on over-representation analysis of the immune system-related pathways redundant among the KEGG, Reactome, and GeneOntology databases (Additional file 1: Table S6) highlights pathways belonging to immune cell communication such as cytokines and chemokines (Fig. 3a). The ssGSEA analysis points out changes in immunological synapse pathways (Fig. 3b). Overall, dysregulation in immune pathways between paired tumor samples was higher in T199LE, T225LE, T227LE, and to a lesser extent T201LE. Several immune checkpoints also showed differential expression between paired samples (Fig. 3c). In particular, a fold change greater than 2 was found in immune checkpoints targeted by immunotherapy, i.e., PDL1 (*CD274*) for T277HP and T278HP, CTLA4 (*CTLA4*) for T227LE, and PD1 (*PDCD1*) for T278HP. In addition, we estimated the relative proportions of immune and stromal populations using MCP-counter defined biomarkers (Additional file 1: Table S2) from RNA-seq data to determine the consistency of the tumor microenvironment between paired samples. Clustering analysis using the estimated cell populations from paired samples integrated with tumor samples from the Bueno and TCGA series [11, 13] separated tumor samples in two clusters, one with a high level of immune cell infiltration corresponding to a hot tumor immune profile, and the other with a lower level of infiltration corresponding to a cold profile (Fig. 4a). Three paired tumor samples from patients T199LE, T225LE, and T227LE, which displayed the highest dysregulation in immune pathways using both pathway analyses, were distributed separately between hot and cold tumors, indicating major changes in terms of the tumor microenvironment composition. Clustering of paired samples without the integration of other tumor samples also separated the tumor paired samples of patients T199LE, T225LE, and T227LE between the cold and hot phenotypes (Additional file 2: Figure S11). These paired samples showed significantly different relative proportions of stromal and immune cell populations with an increase in the infiltration of all cell populations in the hot immune profile paired sample compared to the cold immune profile sample (Fig. 4b). More complex changes with an increase or decrease in certain cell populations between paired samples were also observed in other patients (Additional file 2: Figure S12A). Individual comparison of cell populations showed that natural killer cells, cytotoxic lymphocytes, myeloid dendritic cells, T cells, B cells, and T CD8 cells were variable in paired tumors switching from a cold to a hot immune profile (Additional file 2: Figure S12B). SsGSEA analysis of the aggregated pathways linked to specific immune cell populations confirmed the greatest changes in the infiltration within paired tumors with a hot/cold mixed profile. Comparison between paired tumors with a hot/cold mixed profile and the others displayed significant differences (Mann-Whitney test, *p* = 0.04) for natural killer cells, myeloid dendritic cells, and T cells (Additional file 2: Figure S13). IHC using anti-CD3, anti-CD8, and anti-CD20 antibodies on 3 FFPE tumor samples per patient, biopsied at distant anatomic sites, showed heterogeneous infiltration of T and B cells in T199LE patient and to a lesser extent in T225LE and T227LE patients (Additional file 2: Figure S14).

## Discussion

The present study provides a comprehensive description of spatial intra-tumor heterogeneity in MPM, along with new insights into tumor evolution and clues to the impact on therapy.

By a robust analysis of clonality restricted to validated somatic protein-altering variants, we confirmed the occurrence of MPM populations with different mutation profiles, depending on the anatomic site in the same patient, as previously observed [17, 18]. All tumors harbored many shared clonal mutations, supporting the notion that MPM was derived from a single cell in our series, contrary to the polyclonal origin previously described [15]. Along with these shared clonal variants, the presence of private clonal or subclonal variants indicates that tumor cells spread in the thoracic cavity and continue to evolve separately at different locations. One important finding was the existence of subclonal tumor populations shared between anatomic sites for at least one patient and likely in three others, which supports polyclonal dissemination throughout the thoracic cavity in some MPM patients. Evidence of polyclonal dissemination has been observed in metastases for several cancers [46]. This is possibly related to multiple waves of migrating cells or to the implantation of circulating tumor cell clusters composed of genetically distinct clones that have been described in biological fluids [47]. For MPM, numerous large tissue fragments and multicellular balls or berry-like clusters of cells are observed by cytology in the pleural fluid of patients [48, 49]. Our results suggest that these multicellular structures of tumor cells participate in the spread of MPM in the thoracic cavity.

*NF2* is one of the main mutated tumor suppressor genes in MPM. As it plays a key role during mesothelial carcinogenesis, inactivating variants were expected to be clonal [50]. However, we clearly demonstrate the presence of subclonal *NF2* mutations in three patients, characterized by clonal mutation in other key MPM mutated cancer genes including *BAP1*, *SETD2*, and the *TERT* promoter. The existence of MPM subclones harboring *NF2* mutations was first suggested by a study identifying one cell line with homozygous *NF2* mutation, although this was almost undetectable in the original tumor in a series of nine MPM patients [16]. Heterogenous *NF2* mutations between different tumor regions were also reported in two out of nine patients in another study, but the subclonality was not confirmed by a rigorous CCF analysis as we performed here [18]. Altogether, these results suggest that subclonal *NF2* mutations are frequent in MPM and support that *NF2* inactivation could be a late event during MPM development. Merlin encoded by the *NF2* gene, is a multifunctional protein exhibiting well-established tumor-suppressive function through several cellular processes, not only linked to the activation of Hippo pathway, but also to the inhibition of PI3K/AKT/mTOR pathway as well as to regulatory functions in the nucleus [50]. Although *NF2* inactivation is clearly a driver of mesothelial carcinogenesis, it is possible that this inactivation does not play a role in tumor initiation for MPM, but will promote the development of a more aggressive tumor. This hypothesis is in line with several findings: (i) inactivation of *NF2* in mouse models led to a variety of malignant tumors but not to mesothelioma [51], except if this inactivation was associated with asbestos exposure or with the inactivation of other tumor suppressor genes [52]; recent studies screening germline mutations in large cohorts of patients (reviewed in [44]) did not identify *NF2* as a cancer susceptibility gene for MPM; and (iii) *NF2* mutations showed a significantly higher mutation rate in MPM with an advanced stage [22]. We previously showed that MPM with mutations in members of the Hippo pathway could be more sensitive to specific anti-cancer molecules [53]. The Hippo pathway is becoming attractive for targeted therapy in cancer, and numerous companies are developing compounds to inhibit the Hippo pathway [54]. Implementation of a therapeutic strategy based on Hippo pathway dysregulation will need to take into account the clonality of *NF2* mutations in MPM. A heterogeneous frameshift deletion was also found in *CTNNB1*. Inactivating mutations were previously reported in MPM [22, 55]. Loss of the beta-catenin protein in a MPM subclonal population lead to alterations in the Wnt signaling pathway, which is involved in therapeutic resistance [56]. Consequently, detection of this subclonal population could be of therapeutic interest.

The presence of chromosomal abnormalities including structural changes is a key feature of MPM [57]. However, our data and those of Chen et al. [18] showed that chromosomal profiles are globally similar between different regions within the same tumor. Similarly to copy number aberrations, we did not identify specific relevant gene fusions limited to one of the paired tumor samples. As chromothripsis and chromoplexy lead to massive rearrangements in several chromosome regions in MPM [58, 59], specific omic or NGS approaches will be needed to deeply analyze spatial tumor heterogeneity at the chromosome level. However, our results suggest that the main chromosomal alterations occurring in MPM are early events of mesothelial carcinogenesis, in accordance with the mechanism of action of asbestos, which is well-described as a genotoxic agent inducing chromosomal damage [60].

In addition to the detection of heterogeneous populations characterized by specific mutations, our results highlight major changes at the transcriptomic level between tumors at different anatomic sites in some patients, leading to the dysregulation of specific pathways. We showed that gene expression changes are related to epigenetic mechanisms in at least two patients. Methylation analysis is limited to 5 patients, with only two with consistent changes, and allows only descriptive conclusions to be drawn. Dysregulation of cell adhesion and extracellular matrix organization pathways is frequent in patients and may reflect variations between the proportion of epithelioid and sarcomatoid phenotypes according to the anatomical site. One histologic study showed that a diagnosis of epithelioid MPM in the initial biopsy was changed to the biphasic or sarcomatoid type in 19% of cases when the surgical resection was evaluated [61]. In agreement, we observed variation greater than 10% and up to 23% in the E/S.scores of the paired biopsies of three patients [10]. We found epithelioid tumor samples with high S.score (up to 0.68), consistent with previous observations in larger series [10, 22]. This may be related to the tumor representativity of a FFPE tumor section compared to a piece of frozen tissue, or to a different evaluation sensitivity. Another hypothesis is that the S.score predicts the proportion of sarcomatoid-like cells, which may not have completely a characteristic sarcomatoid cell morphology. Gene expression-based signatures to predict physiological process or prognosis such as the E/S.score or the CV score (*CLDN15*/*VIM*) in MPM [10, 11, 62], as well as response to treatment is becoming popular, and some of them have been evaluated in phase 3 clinical trials for breast cancer [63]. Based on our results, multi-site tumor sampling should be recommended before implementing these assays in MPM.

Finally, we found consequent spatial intra-tumor heterogeneity of the immune microenvironment. We verified that this result was not due to a bias in sequencing depth between samples. This is in line with previous studies showing changes in the T cell repertoires at different anatomic sites [17, 18]. Using the MCP-counter method, whose predictions were validated previously by immunohistochemistry in MPM [10], we emphasized differential immune cell infiltration between paired samples. Importantly, for three patients, the immune profile could be considered hot or cold depending on the paired samples. Substantial gene expression variation was also observed for PDL1, PD1, and CTLA4 in some patients. As immunotherapy with immune checkpoint inhibitors is emerging as a promising therapeutic option for MPM patients, biomarkers to predict the response to this treatment are a crucial issue [2]. No predictive biomarker is currently clearly defined for MPM, but immune cell infiltration and immune checkpoint expression have been suggested in other cancers, indicating that spatial intra-tumor heterogeneity needs to be taken into account to identify biomarkers in MPM [64].

The limitation of this study is the small size of the series in terms of patients and tumor samples per patient. Further studies in larger series are needed to confirm the frequency of our major findings and to have an exhaustive view of spatial intra-tumor heterogeneity in MPM. To predict response to therapy, it would be also crucial to monitor clonal evolution after treatment.

## Conclusions

Spatial intra-tumor heterogeneity is complex in MPM and varies among patients. We highlighted multiple types of heterogeneity, i.e., (i) genetic, (ii) transcriptomic, (iii) epigenetic, and (iv) linked to the immune microenvironment. It was found that the accuracy of histologic classification is increased by the examination of several tumor biopsies, and multi-sampling is recommended [21, 61]. Our molecular analysis also supports the notion that separate anatomic sites should be sampled from the pleural cavity to be able to estimate prognosis or predict response to treatment based on molecular characteristics, with the aim of developing molecular-based precision medicine strategies in order to improve patient survival and quality of life.

## Supplementary Information

**Additional file 1: Table S1.** Clinical annotations of MPM patients. **Table S2.** List of biomarkers included in MCP-counter. **Table S3.** Variants with structural consequences in genes and variants in the *TERT* promoter. **A** Germline mutations identified by whole exome sequencing. **B** Variants correspondence between sequencing methods. **C** Somatic variants with structural consequences in genes and variants in the *TERT* promoter identified by whole exome sequencing. **D** Variants with structural consequences in genes and variants in the *TERT* promoter identified by DNA targeted sequencing. **E** DNA targeted sequencing of multiple sections of the T227LE fresh tumor sample used to generate the MPM_83 primary cell line. **Table S4.** Clonality of the protein-altering somatic variants detected by whole exome sequencing. **A** Cancer cell fraction (CCF) values of protein-altering variants. **B** Clonality per patient based on protein-altering variants. **C** Cancer cell fraction (CCF) values of copy number variations (CNV) based on FACET analysis. **D** Clonality per patient based on FACET analysis. **Table S5.** Fusion transcripts identified by RNA-seq. **Table S6.** Pathway dysregulation between paired tumor samples. **A** Differentially expressed protein coding genes between paired tumor samples. **B** Pathways dysregulated between paired tumor samples in the GeneOntology Biological Process non-redundant database. **C** Pathways dysregulated between paired tumor samples in the KEGG database. **D** Pathways dysregulated between paired tumor samples in the Reactome database. **E** Pathways analysis using single sample Gene Set Enrichment Analysis (ssGSEA) in the GeneOntology Biological Process, KEGG and Reactome databases. **Table S7.** Epigenetic dysregulation between paired tumor samples. **A** Differentially methylated CpG between paired tumor samples. **B** Pathways dysregulated between paired tumor samples based on differentially expressed protein coding genes associated with differentially methylated CpG in the GeneOntology Biological Process non-redundant, KEGG and Reactome databases.

**Additional file 2: Figure S1.** Positron emission tomography (PET) scans of the two patients with biopsies performed on highest metabolite sites. **Figure S2.** Frequencies of *BAP1* and *NF2* variants in the fresh tumor sample used to generate the MPM_83 primary cell line. **Figure S3.** Cancer cell fraction in paired MPM samples. **Figure S4.** Pangenomic copy number variation profile determined by DNAcopy. **Figure S5.** Pangenomic copy number variation profile determined by FACET. **Figure S6.** Fusion transcripts identified by RNA-seq. **Figure S7.** Unsupervised clustering of MPM tumor samples based on gene expression profiles. **Figure S8.** Differentially expressed genes between paired tumor samples. **Figure S9.** Single sample Gene Set Enrichment Analysis (ssGSEA) for pathways related to cell adhesion and extracellular matrix. **Figure S10.** Epigenetic intra-tumor heterogeneity. **Figure S11.** Differential infiltration of stromal and immune cell populations. **Figure S12.** Distribution of immune and stromal populations among paired tumor samples. **Figure S13.** Single sample Gene Set Enrichment Analysis (ssGSEA) for pathways related to immune cell infiltration. **Figure S14.** Immunohistochemical staining of tumor-infiltrating T and B cells in patients with paired tumor samples switching from a cold to a hot immune profile.

## Data Availability

The datasets supporting the conclusions of this article are included within the article (and its additional files). The NGS data (Targeted NGS, whole exome sequencing, RNA-Seq) are available in the European Genome-phenome Archive (EGA) under the EGAS00001005328 study number, https://ega-archive.org/studies/EGAS00001005328 [23]. The methylome data are available in Gene Expression Omnibus (GEO) repository under the GSE175769 series number, https://www.ncbi.nlm.nih.gov/geo/query/acc.cgi?acc=GSE175769 [38].

## Abbreviations

CCF: Cancer cell fraction
DE: Differentially expressed
DM: Differentially methylated
MPM: Malignant pleural mesothelioma
NGS: Next-generation sequencing
PET: Positron emission tomography
ssGSEA: Single-sample Gene Set Enrichment Analysis
TERT_prom: TERT promoter
WES: Whole exome sequencing
