# Supplementary material for: Multi-site tumor sampling highlights molecular intra-tumor heterogeneity in malignant pleural mesothelioma
Source: Genome Med. 2021 Jul 14;13:113. doi: 10.1186/s13073-021-00931-w (PMC8281651; doi:10.1186/s13073-021-00931-w)
Supplement: Supplementary file 2 — Additional file 2: Figure S1. Positron emission tomography (PET) scans of the two patients with biopsies performed on highest metabolite sites. Figure S2. Frequencies of BAP1 and NF2 variants in the fresh tumor sample used to generate the MPM_83 primary cell line. Figure S3. Cancer cell fraction in paired MPM samples. Figure S4. Pangenomic copy number variation profile determined by DNAcopy. Figure S5. Pangenomic copy number variation profile determined by FACET. Figure S6. Fusion transcripts identified by RNA-seq. Figure S7. Unsupervised clustering of MPM tumor samples based on gene expression profiles. Figure S8. Differentially expressed genes between paired tumor samples. Figure S9. Single sample Gene Set Enrichment Analysis (ssGSEA) for pathways related to cell adhesion and extracellular matrix. Figure S10. Epigenetic intra-tumor heterogeneity. Figure S11. Differential infiltration of stromal and immune cell populations. Figure S12. Distribution of immune and stromal populations among paired tumor samples. Figure S13. Single sample Gene Set Enrichment Analysis (ssGSEA) for pathways related to immune cell infiltration. Figure S14. Immunohistochemical staining of tumor-infiltrating T and B cells in patients with paired tumor samples switching from a cold to a hot immune profile. [file 13073_2021_931_MOESM2_ESM.pdf]

A

T225LE

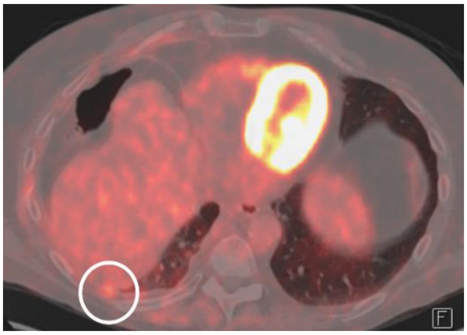

	SUV peak
**Apex**	< 2.5
**Highest metabolic site (Costo-diaphragmatic)**	2.9

B

T227LE

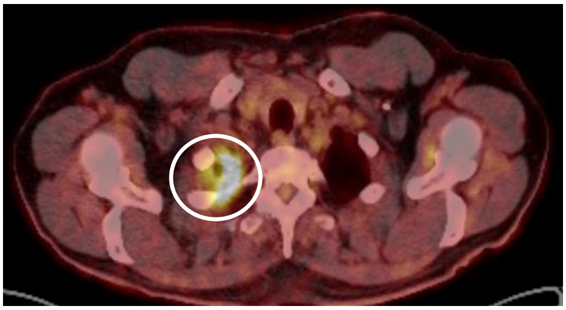

	SUV peak
**Apex**	5-7.5
**Highest metabolic site (Apex)**	>10

Fig. S1 Positron emission tomography (PET) scans of the two patients with biopsies performed on highest metabolite sites.

The 18F-fluorodeoxyglucose PET scans were performed after diagnostic videothoracoscopy and before curative surgery (extended pleurectomy decortication). On the right, cross-sections of a PET scan of patients T225LE (**A**) and T227LE (**B**), focusing on the costo-diaphragmatic biopsy area and on the apical biopsy area, respectively, are shown. Metabolic areas are in yellow and non metabolic area are not in color. The white circles indicate the areas of the biopsies performed on highest metabolite sites. On the left, the tables show the standardized uptake values (SUV) peak (g/ml), measured with the same PET scanner, of the areas of both paired tumor biopsies for each patient.

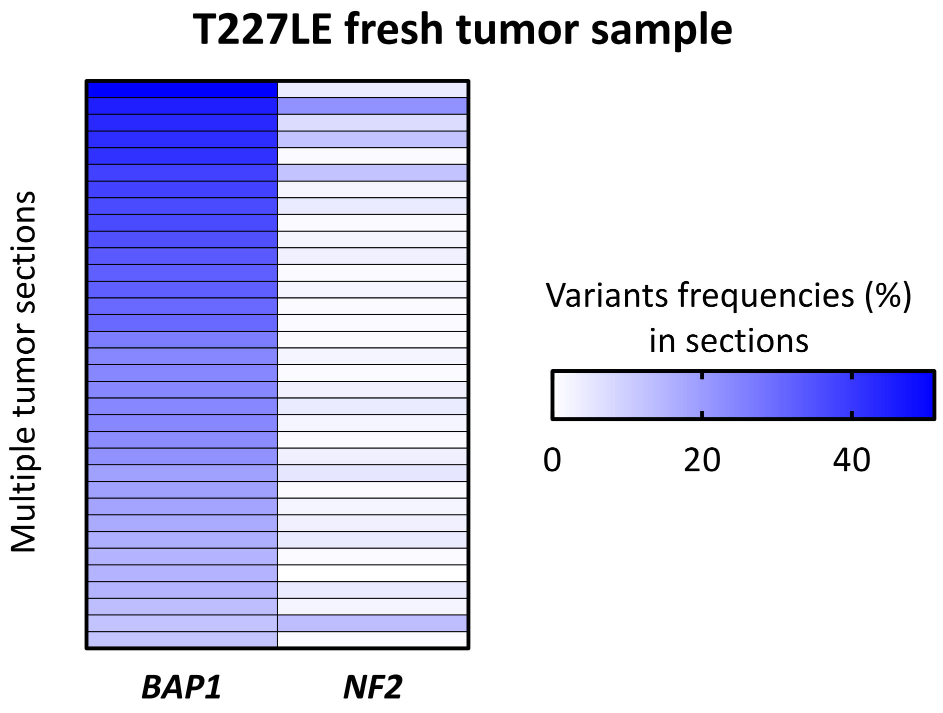

Fig. S2 Frequencies of *BAP1* and *NF2* variants in the fresh tumor sample used to generate the MPM_83 primary cell line.

Frequencies of *BAP1* (chr3:52437896delC) and *NF2* (chr22:30057302C>T) variants are shown in the heatmap, obtained after sequencing of 34 sections of the fresh tumor sample used to generate the MPM_83 primary cell line. Sections are sorted from the highest *BAP1* variant frequency (dark blue) to the lowest (light blue). These results confirm the presence of the *NF2* subclonal variant in the tumor sample.

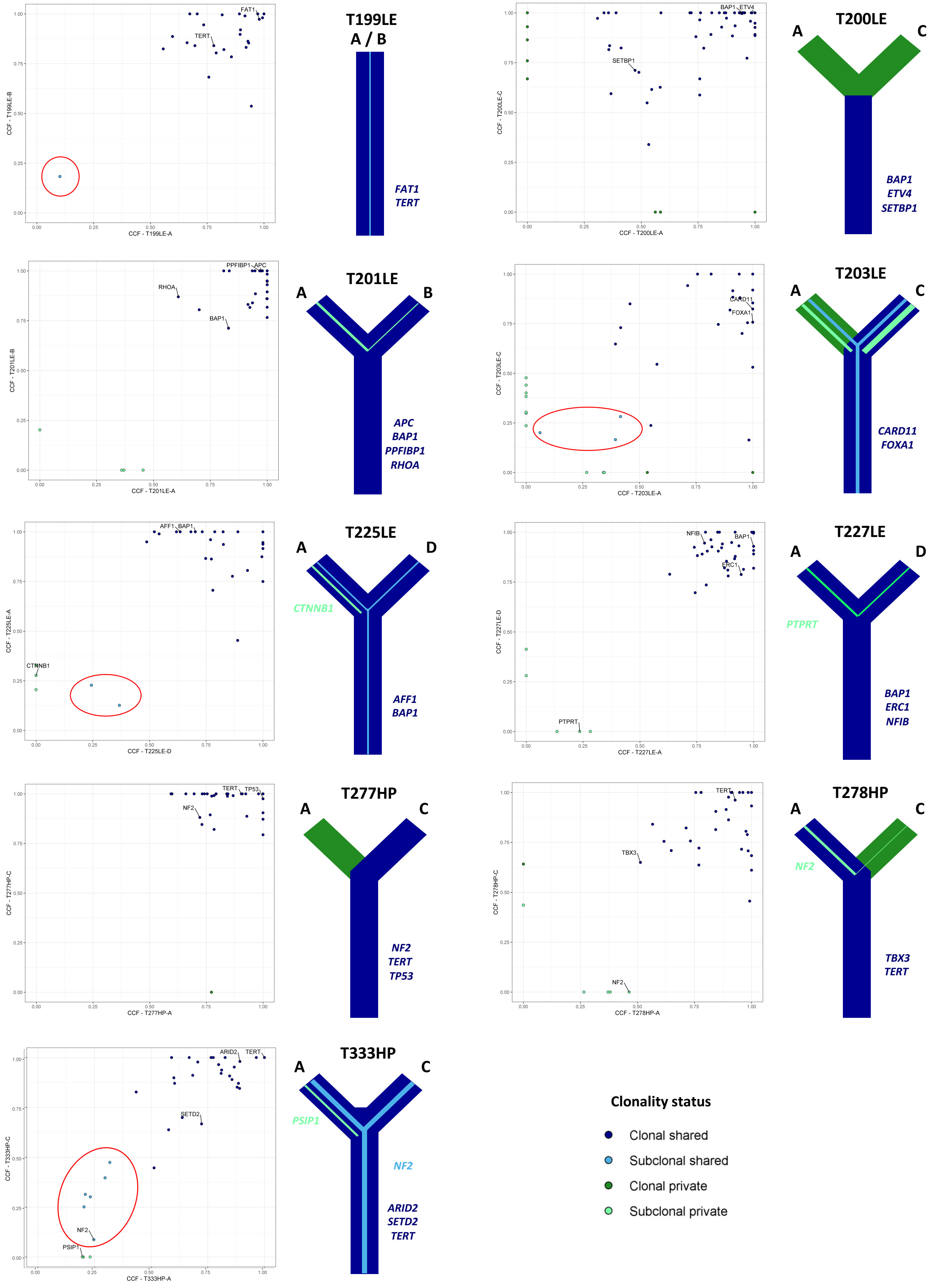

Fig. S3 Cancer cell fraction in paired MPM samples.

In the left graph, each dot represents the adjusted cancer cell fraction value of a given variant in both paired biopsies. Each variant was then categorized according to its clonality (clonal when present in all cells of a sample) as well as to its spatial segregation (shared when present in two biopsies, private otherwise) and was colored according to its clonality status. Cancer-related genes are indicated. Subclonal variants shared among paired biopsies are highlighted with a red circle and might be consistent with the polyclonal diffusion of these tumors throughout the thoracic cavity. On the right, a tree schematically illustrates the clonal evolution of the tumor. For subclonal populations, the line width is proportional to the number of variants.

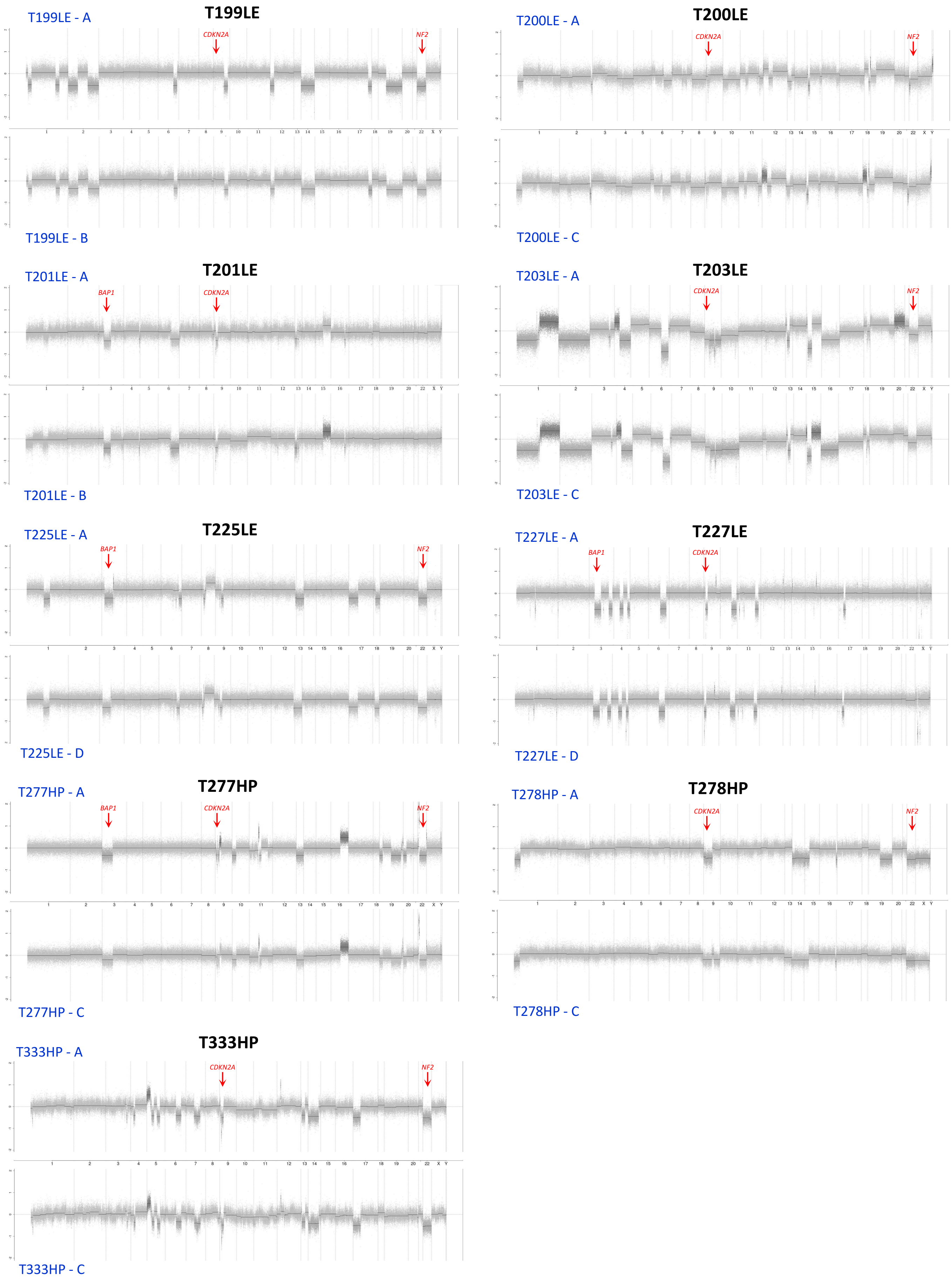

Fig. S4 Pangenomic copy number variation profiles determined by DNAcopy.

Each graph represents the copy number variations (log ratio) in a tumor sample compared to the non-tumoral paired sample for the single nucleotide variations known in the 1000 Genomes Project along the genome (chromosomes sorted on the x axis). Copy number variation profiles of tumor samples from the same patient are grouped in order to facilitate comparison among them. The loci of *CDKN2A*, *BAP1* and *NF2* are indicated by a red arrow when copy number variations were detected.

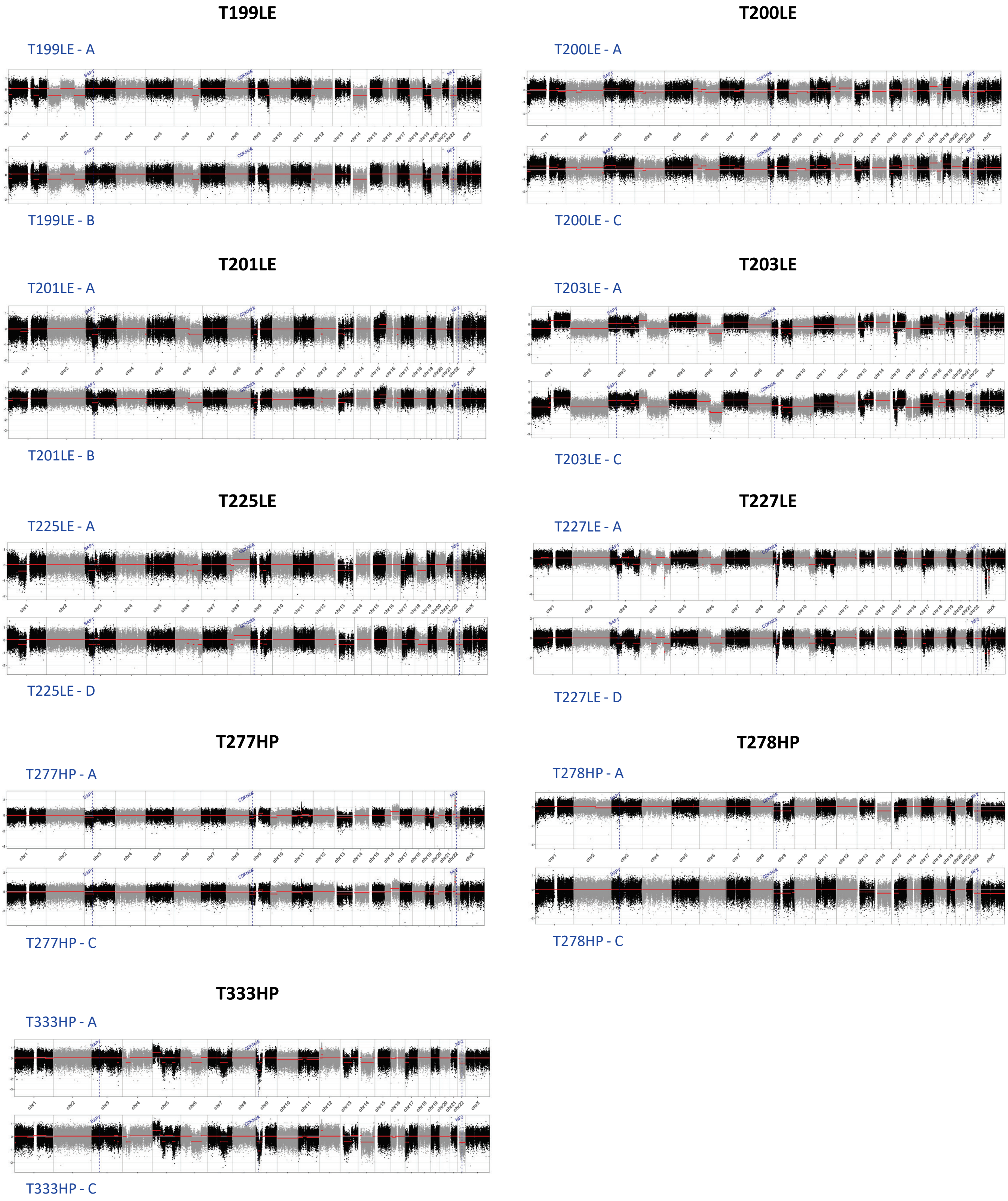

Fig. S5 Pangenomic copy number variation profiles determined by FACET.

Each graph represents the copy number variations (log ratio) in a tumor sample compared to the non-tumoral paired sample (chromosomes sorted on the x axis). Copy number variation profiles of tumor samples from the same patient are grouped in order to facilitate comparison among them. The loci of *BAP1, CDKN2A* and *NF2* are indicated by a dashed blue line.

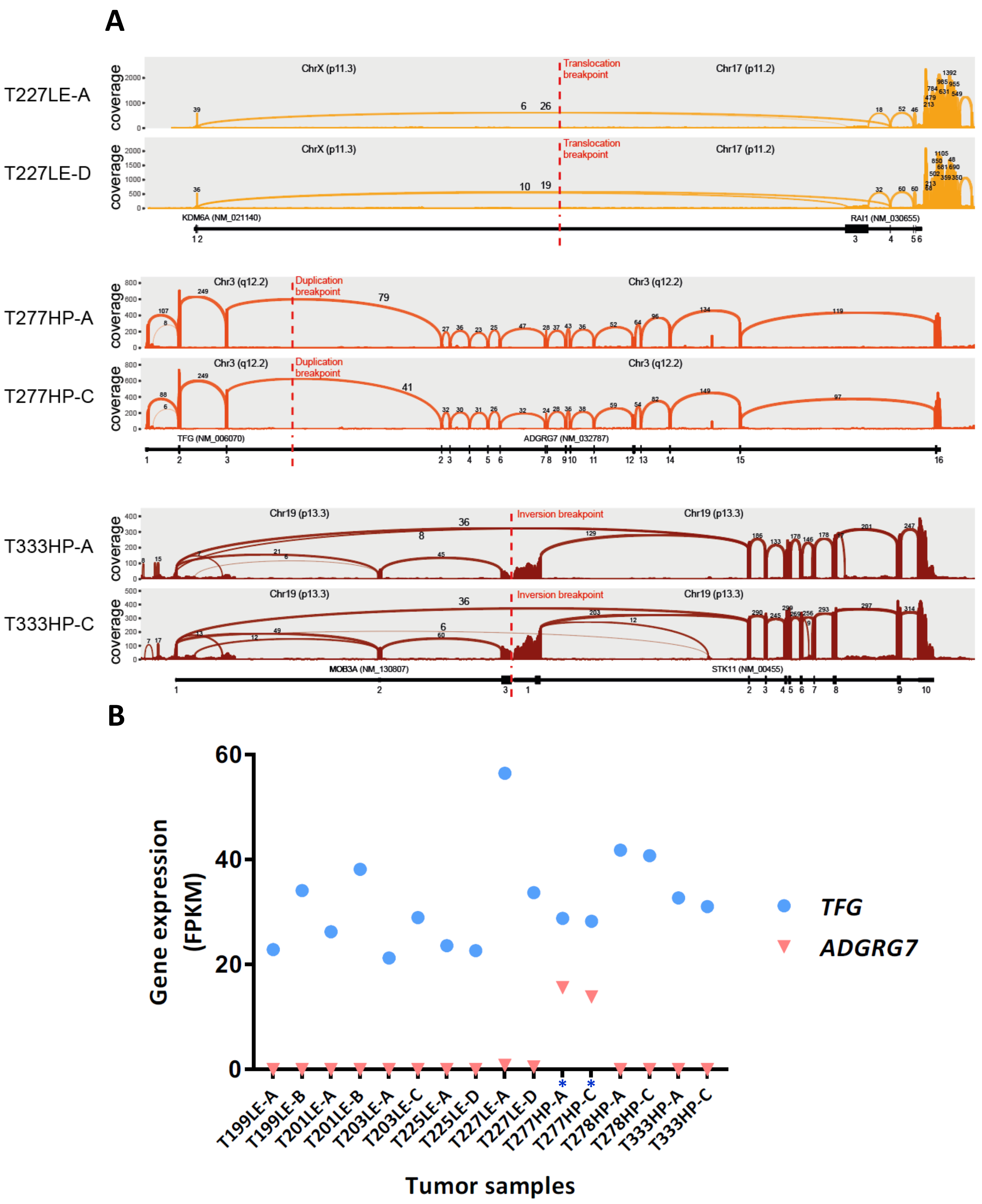

Fig. S6 Fusion transcripts identified by RNA-seq.

A Fusion transcripts involving cancer-related genes. Sashimi plots show RNA-seq alignments for the *KDM6A-RAI1*, *ADRG7-TFG* and *MOB3A-STK11* fusion transcripts identified in T227LE, T277HP and T333HP paired tumor samples, respectively. Splice junction reads are represented as arcs connecting a pair of exons. The arc width is proportional to the number of reads aligning to the junction and the number of supporting reads is indicated. The chromosome position of each gene and the type of fusion are indicated at the top in black and red, respectively. **B** *ADGRG7* gene expression induced by the fusion transcript. Blue dots and red triangles represent *TFG* and *ADGRG7* gene expression (FPKM), respectively, in the series of tumor samples analyzed by RNA-seq. The paired tumor samples expressing the fusion transcript involving *TFG* and *ADGRG7* are indicated by a blue asterix. TSG: tumor suppressor gene.

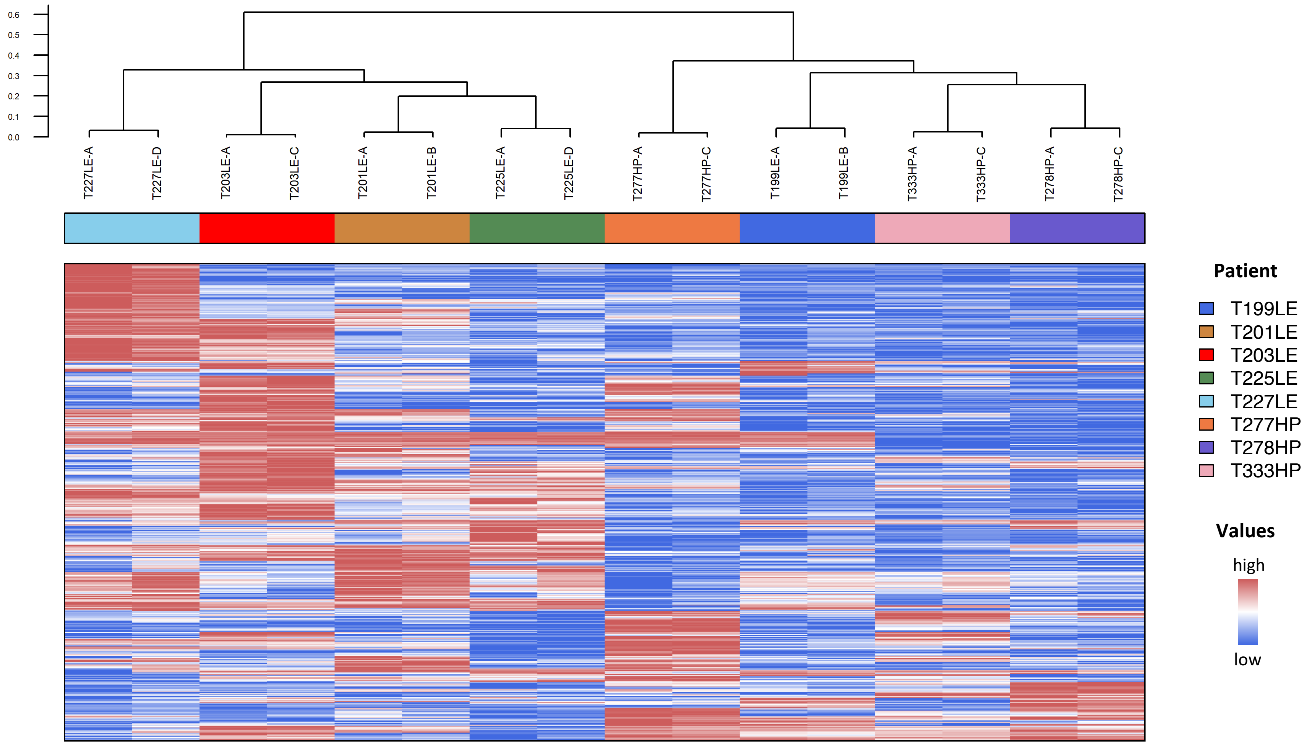

Fig. S7 Unsupervised clustering of MPM tumor samples based on gene expression profiles.

The dendrogram obtained after clusterization of MPM tumor cells on the 500 most differentially expressed genes shows that each paired tumor sample clustered together.

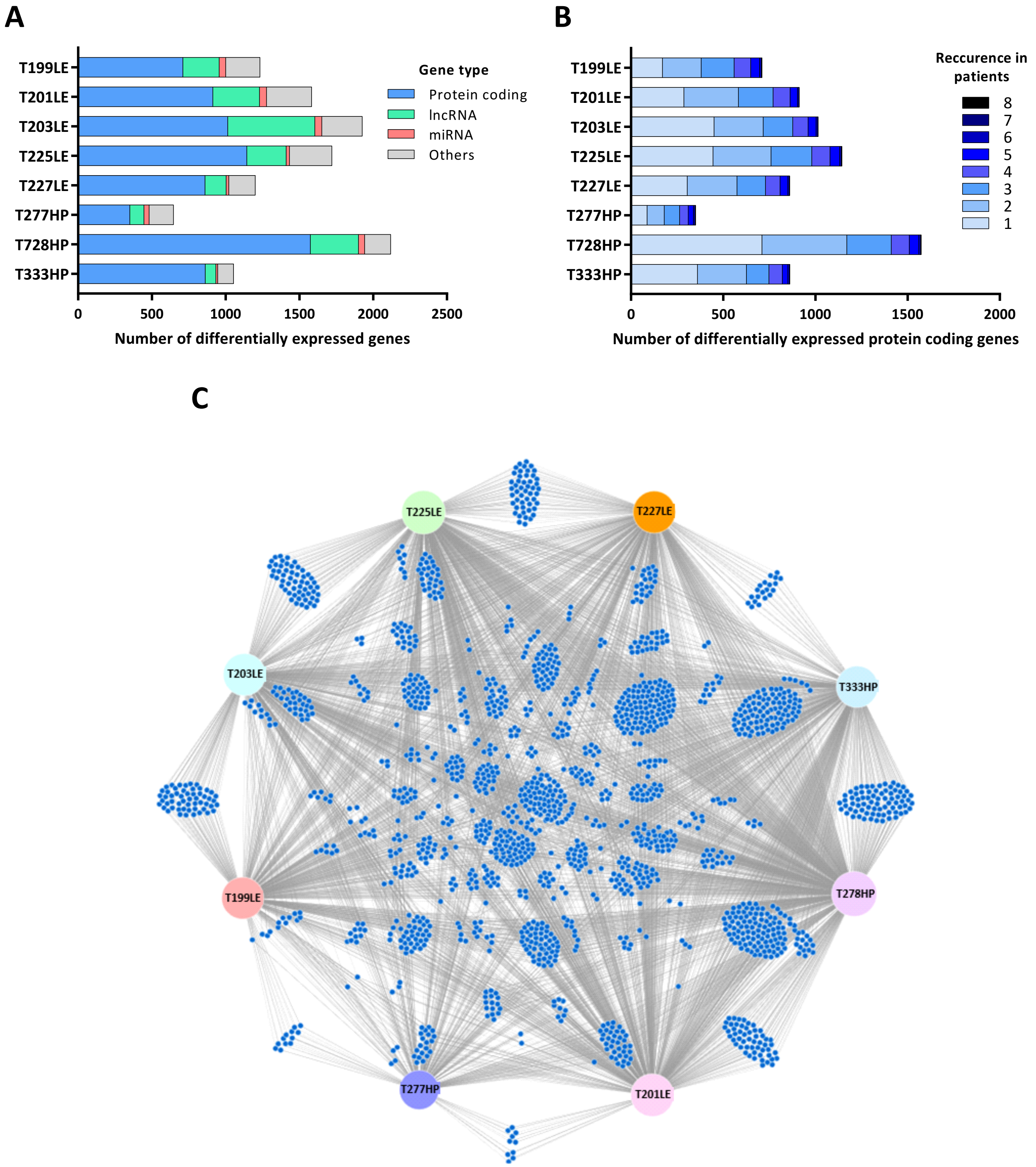

Fig. S8 Differentially expressed genes between paired tumor samples.

A The histogram shows the number of differentially expressed genes per tumor sample and indicates the biotypes of these genes using color coding. **B** The histogram shows the number of differentially expressed protein coding genes per tumor sample and highlights by a blue color gradient the proportion of these genes found in one to eight patients. **C** The network displays the protein coding genes found differentially expressed in at least two tumor samples. Each circle corresponds to a tumor sample, and each blue dot is a gene linked by a gray line to the tumor samples in which the gene was found to be differentially expressed. lncRNA: long non-coding RNA; miRNA: microRNA.

A

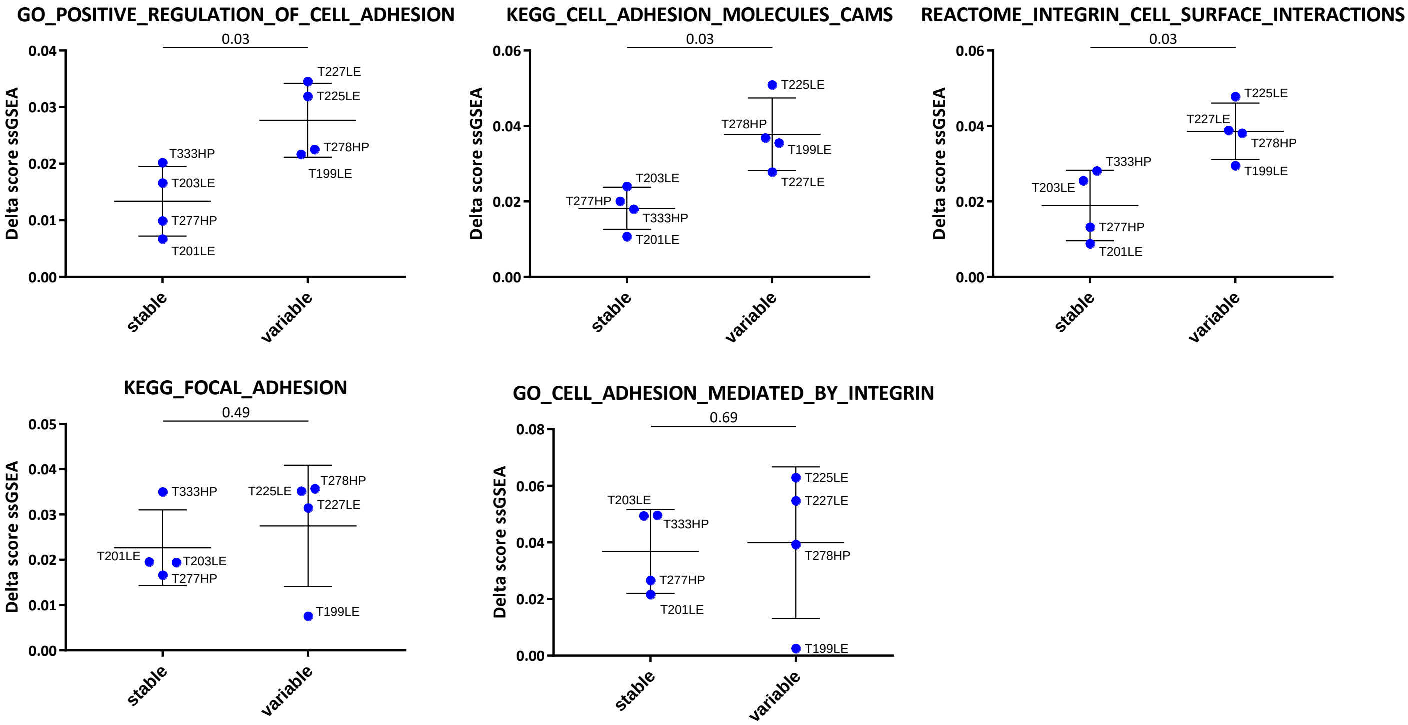

B

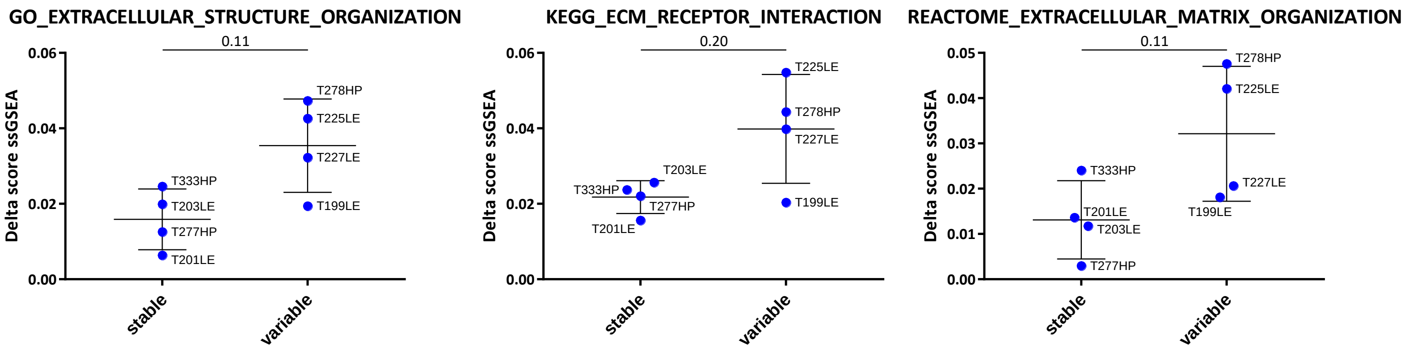

Fig. S9 Single sample Gene Set Enrichment Analysis (ssGSEA) for pathways related to cell adhesion and extracellular matrix.

The differences in the ssGSEA score (delta_score) of each paired tumor sample are shown in the dot point graphs in two groups based on over-representation analysis (Fig. 2a): stable patients (T201LE, T203LE, T277HP and T333HP) and variable patients (T199LE, T225LE, T227LE and T278HP) for the reported pathways related to cell adhesion (**A**), and to extracellular matrix (**B**). The pathway "Integrin activation" was not included in the ssGSEA analysis. The p-values of the Mann Whitney test comparing the distribution between stable and variable patients are indicated at the top of the graph.

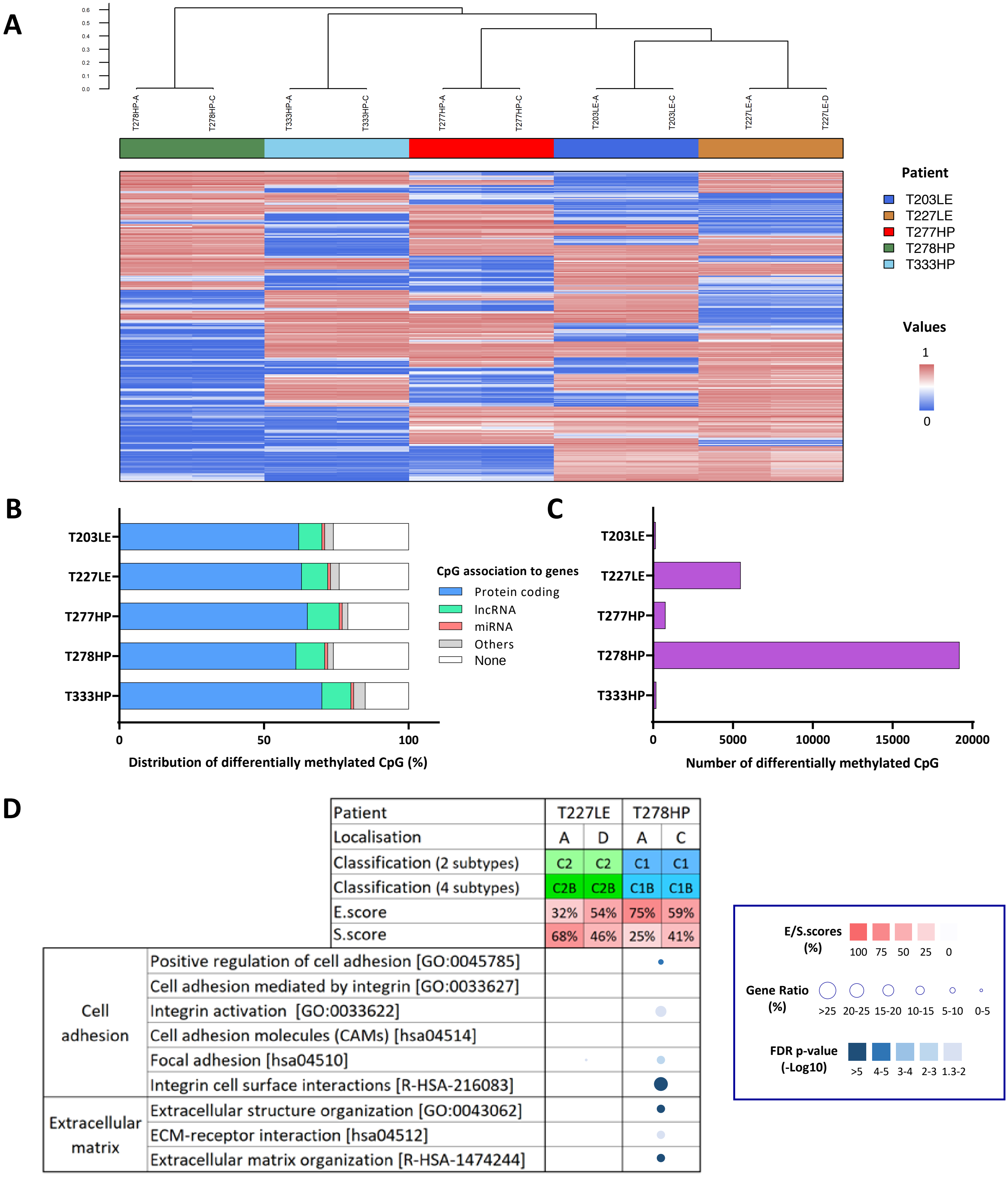

Fig. S10 Epigenetic intra-tumor heterogeneity.

A Unsupervised clustering of MPM tumor samples was performed based on DNA methylation profiles. The dendrogram obtained after clusterization of MPM tumor cells on the 500 CpG showing the most variability in their methylation beta-value highlights that each paired tumor sample clustered together. **B** The histogram shows the distribution of differentially methylated CpG per tumor sample according to the association of the CpG to the loci of genes coding for proteins, lncRNA, miRNA and other biotypes of genes. lncRNA: long non-coding RNA; miRNA: microRNA. **C** The histogram shows the number of differentially methylated CpG per tumor sample. **D** Over-represented pathways linked to cell adhesion and the extracellular matrix among the differentially expressed protein coding genes associated with differentially methylated CpG between paired tumor samples are shown in the table. For each patient, the tumor location is indicated (A: apex; B: side wall; C: costo-diaphragmatic; D: highest metabolic site). The molecular classifications into two to four subtypes and the E/S.scores were predicted based on RNA-seq data and are colored in blue or green depending on the subtypes and with a red gradient for the E/S.scores. Pathway over-representation is indicated by a circle with a size and a color proportional to the gene ratio and the FDR p-values, respectively.

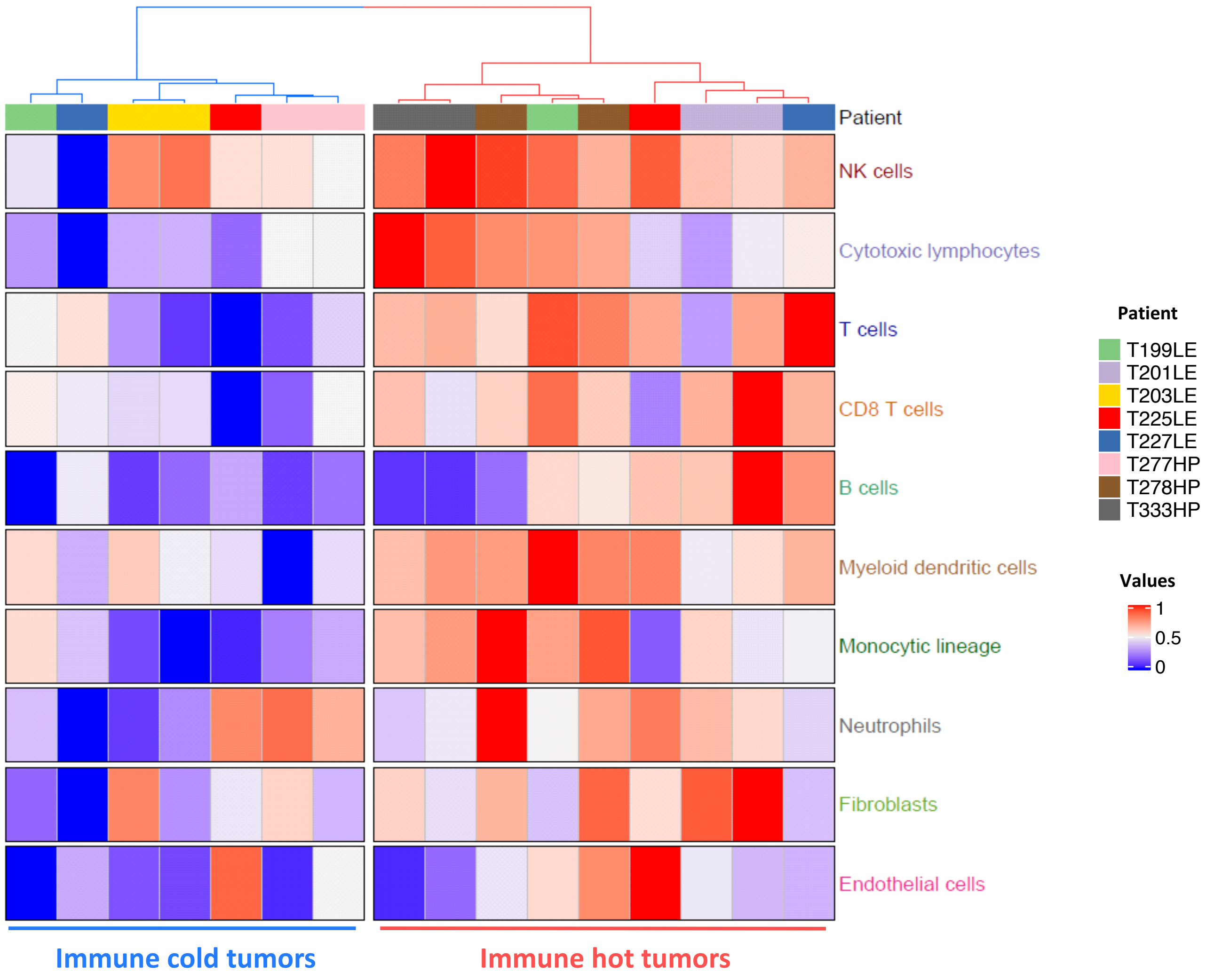

Fig. S11 Differential infiltration of stromal and immune cell populations.

Unsupervised clustering of the paired tumor samples of eight patients was performed based on cell populations determined by the MCP-counter method and is shown as a heatmap.

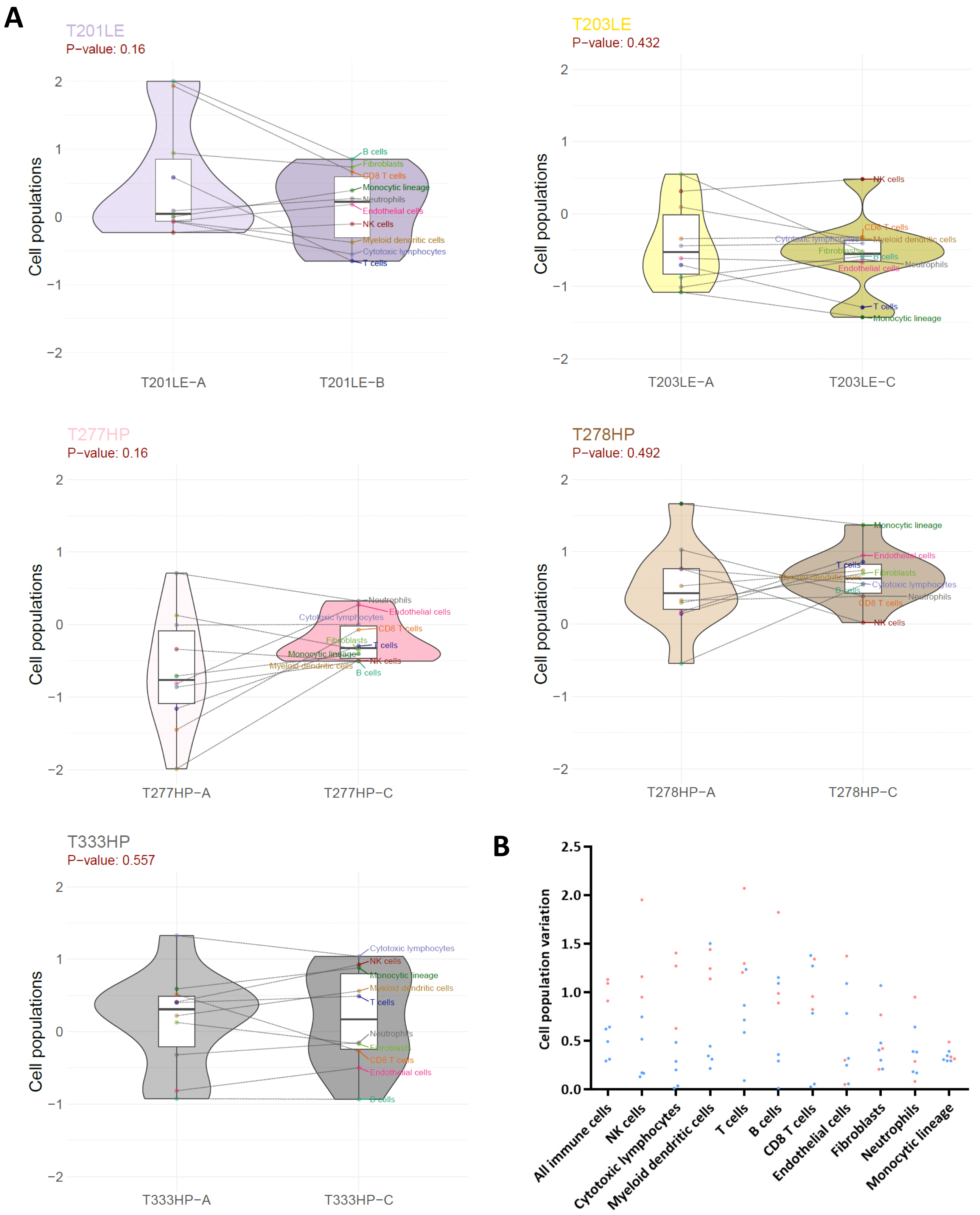

Fig. S12 Distribution of immune and stromal populations among paired tumor samples.

A The violin plots show the normalized MCP-counter values of immune and stromal cell populations between paired tumor samples for patients without a hot/cold mixed profile. Each cell population is indicated in the box plots by a color point connected by a line between paired tumor samples. The p-values of the Mann Whitney test comparing the distribution between paired tumor samples are indicated at the top of the violin plot. **B** The variation of the normalized MCP-counter values are represented for each cell population with red points for paired tumors with a hot/cold mixed profile and blue points for the others. For all immune cells, the mean of the variations of these cells per tumor was calculated.

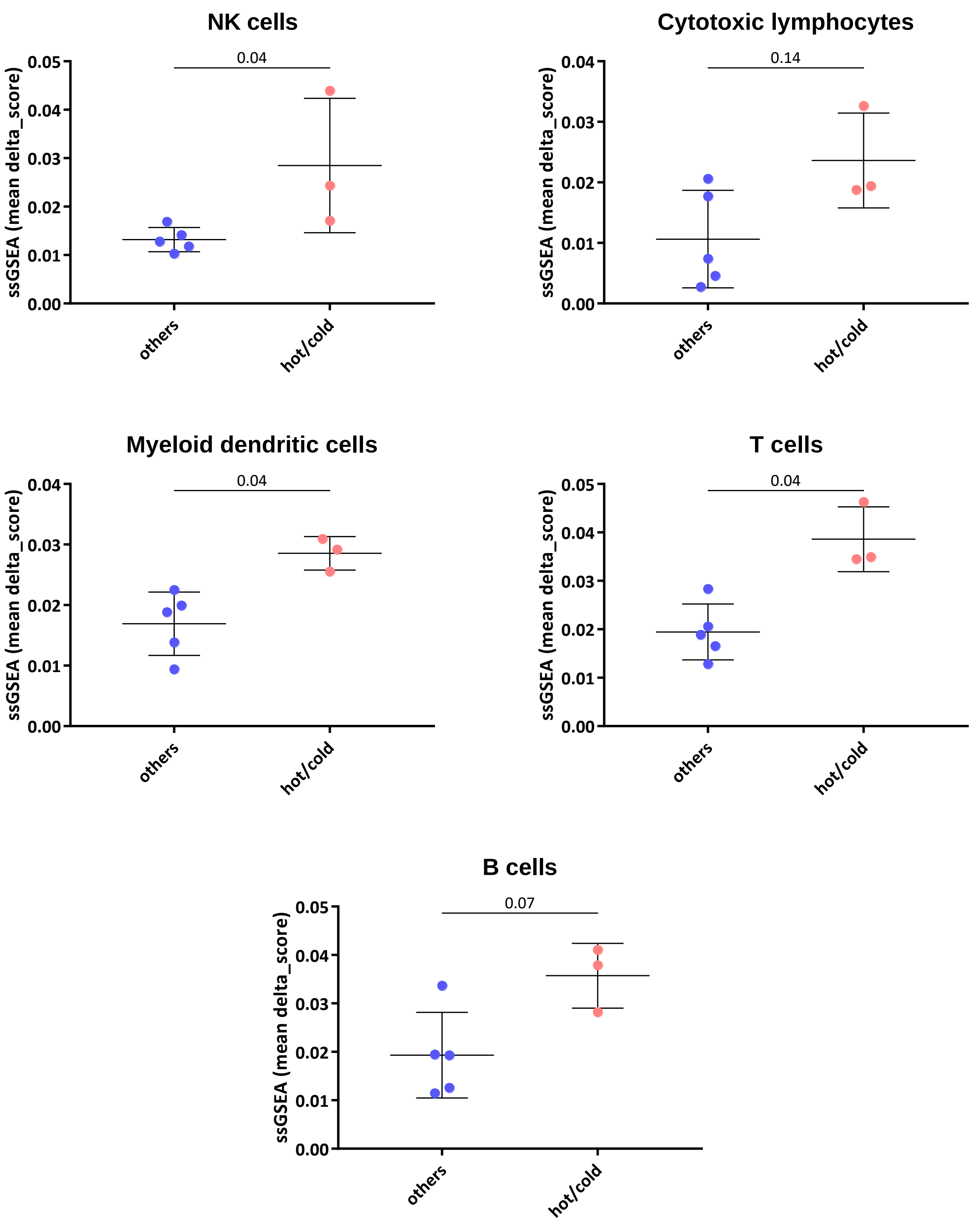

Fig. S13 Single sample Gene Set Enrichment Analysis (ssGSEA) for pathways related to immune cell infiltration.

The pathways linked to specific immune cell population (reported in "Pathway family" column of the Additional File 1: Table S6E) were aggregated by calculating the mean of the ssGSEA score (delta_score) for each paired tumor samples. The variation in the mean ssGSEA score of each paired tumor samples are shown in the dot point graphs with red points for paired tumors with a hot/cold mixed profile (T199LE, T225LE, T227LE) and with blue points for the others. The p-values of the Mann Whitney test comparing the distribution between both groups are indicated at the top of the graph.

	MCP-Counter	IHC (sample 1)	IHC (sample 2)	IHC (sample 3)
	**anti-CD3**			
**T199LE**	-0.05 / 1.15	2+	3+	1+
**T225LE**	-1.52 / 0.55	1+	1+	2+
**T227LE**	0.1 / 1.4	3+	3+	2+
	**anti-CD8**			
**T199LE**	0.01 / 1.35	2+	3+	1+
**T225LE**	-2.08 / -1.13	1+	1+	1+
**T227LE**	-0.17 / 0.65	3+	3+	2+
	**anti-CD20**			
**T199LE**	-1.15 / 0.68	1+	2+	2+
**T225LE**	-0.13 / 0.86	2+	3+	3+
**T227LE**	0.34 / 1.23	1+	1+	2+

Fig. S14 Immunohistochemical staining of tumor-infiltrating T and B cells in patients with paired tumor samples switching from a cold to a hot immune profile.

Immunohistochemistry (IHC) was performed using anti-CD3, anti-CD8 and anti-CD20 antibodies on 3 formalin-fixed, paraffin-embedded (FFPE) tumor samples biopsied at distant sites for T199LE, T225LE and T227LE patients. The MCP-counter scores of paired frozen tumor samples and the IHC staining scores for the 3 FFPE tumor samples are indicated in the table for each patient.
